# Comprehensive assessment of occupational exposure to microbial contamination in waste sorting facilities from Norway

**DOI:** 10.3389/fpubh.2023.1297725

**Published:** 2023-12-19

**Authors:** Carla Viegas, Elke Eriksen, Bianca Gomes, Marta Dias, Renata Cervantes, Pedro Pena, Elisabete Carolino, Magdalena Twarużek, Liliana Aranha Caetano, Susana Viegas, Pål Graff, Anani Komlavi Afanou, Anne Straumfors

**Affiliations:** ^1^H&TRC – Health & Technology Research Center, ESTeSL – Escola Superior de Tecnologia e Saúde, Instituto Politécnico de Lisboa, Lisbon, Portugal; ^2^NOVA National School of Public Health, Public Health Research Centre, Comprehensive Health Research Center, CHRC, NOVA University Lisbon, Lisbon, Portugal; ^3^National Institute of Occupational Health (STAMI), Oslo, Norway; ^4^CE3C – Center for Ecology, Evolution and Environmental Change, Faculdade de Ciências, Universidade de Lisboa, Lisbon, Portugal; ^5^Kazimierz Wielki University, Faculty of Biological Sciences, Department of Physiology and Toxicology, Chodkiewicza, Bydgoszcz, Poland; ^6^Research Institute for Medicines (iMed.uLisboa), Faculty of Pharmacy, University of Lisbon, Lisbon, Portugal

**Keywords:** occupational exposure assessment, microbial agents, manual and automated waste sorting, azole resistance screening, *Aspergillus* spp.

## Abstract

**Introduction:**

It is of upmost importance to contribute to fill the knowledge gap concerning the characterization of the occupational exposure to microbial agents in the waste sorting setting (automated and manual sorting).

**Methods:**

This study intends to apply a comprehensive field sampling and laboratory protocol (culture based-methods and molecular tools), assess fungal azole resistance, as well as to elucidate on potential exposure related health effects (cytotoxicity analyses). Skin-biota samples (eSwabs) were performed on workers and controls to identify other exposure routes.

**Results:**

In personal filter samples the guidelines in one automated industry surpassed the guidelines for fungi. Seasonal influence on viable microbial contamination including fungi with reduced susceptibility to the tested azoles was observed, besides the observed reduced susceptibility of pathogens of critical priority (Mucorales and *Fusarium* sp.). *Aspergillus* sections with potential toxigenic effect and with clinical relevance were also detected in all the sampling methods.

**Discussion:**

The results regarding skin-biota in both controls´ and workers´ hands claim attention for the possible exposure due to hand to face/mouth contact. This study allowed concluding that working in automated and manual waste sorting plants imply high exposure to microbial agents.

## 1 Introduction

There is still much to investigate to fill the knowledge gap regarding the most suitable protocols (from the field to the lab) to assess occupational exposure to microbiological agents and to conclude about the potential health risks in each occupational environment (1, 2). One of the most challenging occupational environment is the waste sorting industry (3, 4) due to several reasons: (a) the waste materials can serve as substrate and provide the needed nutrients for the microorganisms’ proliferation (5); (b) the dust generated in all the workplaces can be a perfect vehicle for the microbial contamination dissemination and reach the workers respiratory tract (3, 6, 7); (c) the waste handling (domestic triage, transport duration, …) before reaching a sorting unit can vary greatly among city, region and country and this will affect the microbial contaminants in the waste and, consequently, the workers’ exposure (8). These variables, among others, remain to have influence on workers’ exposure to microbial contaminants even in modern automated waste sorting plants (9); (d) and the fact that this occupational environment has been reported as a hot spot for two emergent occupational risks needed to be fully addressed: mycotoxins (3, 7) and fungal azole resistance (2, 3, 10).

Waste management industries, and more specifically the ones dedicated to sorting waste, are critical to achieve the Sustainable Development Goals (SDGs) proposed by World Health Organization. Ever since the European Union’s (EU) approval of the Circular Economy (CE) action plans in 2015, expectations toward the waste sorting industries to meet the CE principles have been high (11). To accomplish this endeavor the number of waste sorting facilities and respective workforce is expected to increase in all the EU countries and partners. As the European Economic Area agreement grants Norway access to the EU’s single market the need to achieve these principles will be of upmost importance also for this country.

Norwegian employers are subjected to national regulation that implies the assessment and prevention of exposure to occupational risks (12) and specifically to biologic agents (12). Although there is scientific evidence that associates occupational exposure to microbial agents (bacteria and fungi) to health outcomes (1, 13–15), the health risks due to occupational exposure to microorganisms are frequently less recognized and underreported when compared to chemical exposures (2). In addition, exposure-related health effects on the respiratory tract have been reported in waste workers (16, 17). Indeed, previous studies already concluded that the waste management setting implies high exposure to microorganisms (2, 8, 9, 18). However, exposure determinants, characteristics of the determinants and the possible health effects related still need to be fully unveiled. In this study we intend to complement the findings obtained in previous studies (9, 19–21) and to contribute to fill the knowledge gap regarding occupational exposure to microbial agents in the waste sorting setting (performed automatically and manually) applying a novel and comprehensive field (active and passive sampling methods) and laboratory protocol (culture based-methods and molecular tools). This will allow to understand if the type of sorting influence the microbiological contamination and main features. Furthermore, this study aimed to assess fungal azole resistance, as well as to elucidate potential exposure related health effects (through cytotoxicity analyses). Skin-biota samples were also performed on workers and controls to identify other exposure routes besides inhalation.

## 2 Materials and methods

### 2.1 Waste sorting plants characterization

**Figure 1:**
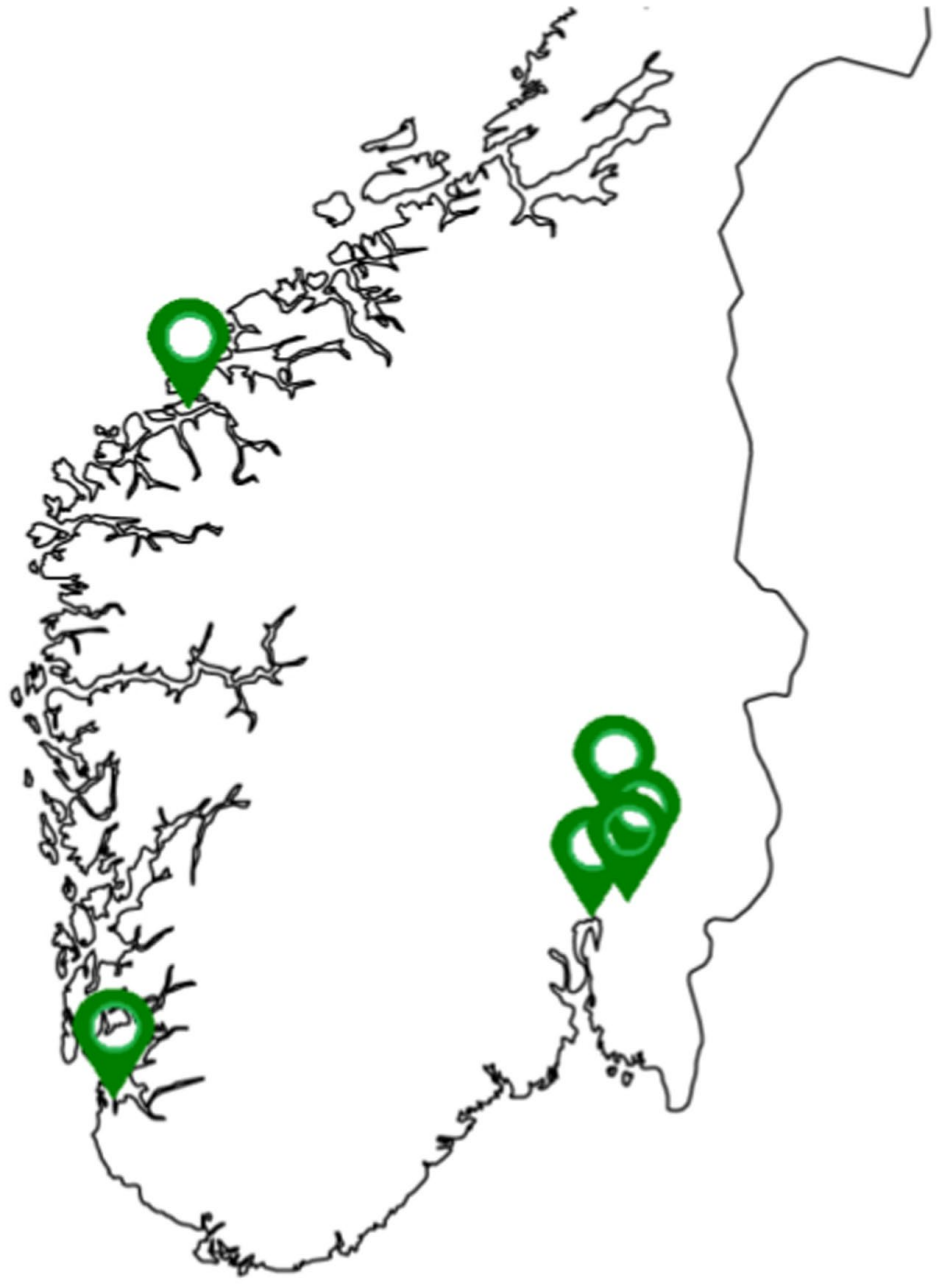
Geographical distribution of the waste sorting plants considered.

The sampling campaign of waste sorting plants occurred between June 2020 and November 2021. Three manual (private companies) and three automated (inter-municipal) waste sorting plants enrolled in the study were assessed, in western and eastern Norway (Figure 1).

Waste sorting differed among plants. In manual plants, primarily pre-sorted waste from housing collectives and local businesses was treated. Plastic and paper/cardboard waste was sorted by hand (with valuable material being returned to the value chain), whereas residual waste was sorted by excavators, shredded and transported to incineration plants. Regarding the work tasks performed, manual plant workers performed the same task throughout the workday, every working day. Investigated work operations included manual sorting of plastics and paper/cardboard, controlling incoming waste and driving excavators. In automated waste sorting plants, unsorted residual waste from domestic homes was received, and sorting was achieved by modern, fully automated waste sorting lines that used ballistic separation, air-pressure, and infrared technologies to fractionize the incoming waste. Investigated work operations included control of incoming waste, cleaning and maintenance of sorting machines, supervision of sorting lines from a secluded control room, as well as driving excavators/trucks in waste reception and storage. All plants were visited at least once, one plant was visited twice, and one plant was visited three times.

### 2.2 Workers population involved in skin-biota evaluation

Workers of all the waste sorting facilities enrolled in the study were invited to voluntarily participate in the project. From these companies, a total of 98 participants (73 waste workers – exposed group, 25 offices personal – control group) were enrolled in the study. All controls were office personal from the respective waste sorting plant that at times visited the waste sorting hall. Sample collection happened in the clean zone of each respective sorting plant. Thus, both exposed workers and controls likely had sanitized their hands immediately prior to entering the office area.

Skin-biota samples of the dorsal side of the left hand were collected on day 3 of the sampling campaign (Wednesday) of both exposed workers and the control group. Workers’ hands were “swabbed” right before the lunch break, as they came from the sorting hall.

This study complied with the Helsinki Declaration and Oviedo Convention and all data were stored and analyzed in accordance with the Portuguese General Data Protection Regulation (GDPR) law n° 58/2019. The study was approved by the Regional Committees for Medical research Ethics South East Norway, REK South East (ref. no. 34312). Workers were invited to voluntarily participate in the project and before their enrolment all volunteers filled a written informed consent.

### 2.3 Workplace sampling campaign performed and samples extraction

Personal air-filter samples, impingement samples (Coriolis air sampler), electrostatic dust collectors (EDC), and settled dust were collected throughout nine sampling campaigns over a period of 18 months. The workplaces assessed and the sampling methods used are described in Table 1.

**Table 1:** Workplaces assessed, and sampling methods applied.

Plants	Waste tons sorted/year	Number of exposed workers	Number of contsrols	Workplaces assessed	Samples type and number			
					Air filter samples	Settled dust	Coriolis air sampler	eSwab
Plant A	50,000	29	8	Automated WSP*	24	11	7	22
Plant B	75,000	17	7	Automated WSP	8	8	3	21
Plant C	22,000	7	5	Automated WSP	5	4	0	7
Plant D	140,000	9	2	Manual WSP	6	3	0	0
Plant E	46,000	4	0	Manual WSP	3	3	0	0
Plant F	347,000	7	3	Manual WSP	6	0	0	6

^*^WSP, Waste sorting plant.

Air-filter samples were collected on 25 mm glass fiber filters (pore size 1 μm, GF/A, Whatman, UK) that were mounted in PAS-6 filter cassettes (22). Filter cassettes were attached to air-pumps (GS5200, GSA Messgerätebau GmbH, Germany) and operated at an average air flow of 2 L/min (±10%). The airflow was measured using a Defender 510 (TPF Control B.V., The Netherlands) prior to and after exposure. Filters were extracted for 30 min in sterile conditions with 5 mL NaCl 0.9% + Tween 80 0.05% (250 rpm, room temperature), and stored at −80°C until shipment/analysis (2.5 mL glycerol was added for conservation).

Workplace air samples were collected using a Coriolis μ (Bertin technologies, France). Sterile autoclaved cones were filled with 15 mL sterile filtrated PBS and operated at an air flow of 200 L/min for 10 min. Samples were stored on ice during transport and stored at −80°C until shipment/analysis (2.5 mL glycerol was added for conservation) and used for further molecular detection.

Settled dust was collected using a sterile spatula and stored at −80° C until shipment/analysis. Dust samples were suspended in 0.1% Tween 80 saline (0.9% NaCl) solution (250 rpm, 30 min), using 9.1 mL solution for 1 g of settled dust sample (23).

Electrostatic dust cloths (EDC) were packed under sterile conditions and exposed for 14 days in the workstations. After exposure, the EDCs were returned to The National Institute of Occupational Health in Norway (STAMI) by mail. Upon arrival, EDCs were extracted for 60 min in 20 mL sterile MilliQ water added 0.05% Tween 20 by orbital shaking at 300 rpm at room temperature. Subsequently, eluates were aliquoted, and stored (after glycerol addition) at −80°C until shipment/analysis.

Skin biota samples were collected by swabbing an area of approximately 5 cm^2^ on the dorsal side of the workers hand with circulating motions (for about 10 s). The samples were collected with sterile Copan eSwab 480C regular flocked swab with 1 mL Liquid Amies Medium in Skirted Tube with Plastic White Capture Cap (Copan, Italy). The sampling of skin biota was conducted during work hours. Hand sanitation was performed before samples collection due to strict hygienic measures due to the pandemic. Samples were collected in both the exposed and control group, using the same protocol. The samples were kept refrigerated (0 to 4°C) until arrival at the laboratory, and then frozen at −80°C until further analysis.

### 2.4 Prevalence of cultivable fungi and bacteria

In order to assess the viable microbiota, 150 μL of the prepared sample extracts were inoculated in selective media, as follows: malt extract agar (MEA) supplemented with chloramphenicol (0.05%), and dichloran-glycerol agar (DG18) for fungi (27°C, 5–7 days); tryptic soy agar (TSA) supplemented with nystatin (0.2%) (30°C, 7 days), and Violet Red bile agar (VRBA) (35°C, 7 days) for mesophilic and Gram-negative bacteria, respectively. Microbial quantification was determined as colony-forming units (CFU) and CFU concentration (CFU.m^−3^/m^−2^/m^−2^.day^−1^/g^−1^) depending on the used sampling method. Additionally, fungal species/genera were identified by a trained mycologist through notation of macro and microscopic characteristics (24).

### 2.5 Screening of azole-resistance

In order to address the growing urgency of fungal resistance (25), a preliminary screening of azole-resistance was conducted, as previously reported (26). Briefly, 150 μL of EDC, filter and settled dust samples’ extracts were seeded on azole-supplemented Sabouraud dextrose agar (SDA) media (Frilabo, Maia, Portugal) with final concentrations of 4 mg/L itraconazole (ICZ), 2 mg/L voriconazole (VCZ), and 0.5 mg/L posaconazole (PCZ) [adapted from: Arendrup et al. (27); The European Committee on Antimicrobial Susceptibility Testing (28)]. *A. fumigatus* reference strain (ATCC 204305) and pan-azole-resistant *A. fumigatus* strain (both provided by the National Health Institute Doctor Ricardo Jorge, IP) were used as negative and positive control, respectively. Fungal species/genera were identified after 48 to 72 h’ incubation at 27°C, as described elsewhere (23).

### 2.6 Molecular detection of *Aspergillus* sections

Six important *Aspergillus* sections were targeted in air samples, filter and settled dust samples’ extracts by quantitative PCR (qPCR) using the CFX-Connect PCR System (Bio-Rad), according to a previously reported method (23) and to complement the results already obtained in previous studies (19, 21). For fungal DNA isolation, the ZR Fungal/Bacterial DNA MiniPrep Kit (Zymo Research, Irvine, USA) was used. Reactions were performed in a 20 μL final volume containing 1 × iQ Supermix (Bio-Rad, Portugal), 0.5 μM of each primer, and 0.375 μM of TaqMan probe. qPCR conditions included a three-step reaction consisting of 40 cycles of denaturation at 95°C for 30 s, annealing at 52°C for 30 s, and extension at 72°C for 30 s.

The used controls were water (negative control) and a reference strain DNA (positive control). The reference strains were kindly provided by the reference Unit for Parasitic and Fungal Infections from the Department of Infectious Diseases, National Health Institute Doctor Ricardo Jorge, IP. All reference strains were sequenced for ITS, B-tubulin, and Calmodulin.

### 2.7 Screening for cytotoxicity

In order to assess the toxicological effects of samples collected in the waste sorting plants, human alveolar epithelial (A549) cells and human liver carcinoma (HepG2) cells were exposed to filter (*N* = 18) and settled dust (*N* = 11) samples’ extracts and screened for cytotoxicity.

Firstly, cells were maintained in Eagle’s Minimum Essential Medium (MEM) supplemented with 10,000 units penicillin and 10 mg/mL streptomycin in 0.9% NaCl and fetal bovine serum (Sigma-Aldrich, USA). Then, cells were detached with 0.25% (w/v) Trypsin 0.53 mM EDTA. Cell suspensions (100 μL) with 2.0 × 10^5^ HepG2 cells/ml and 4.7 × 10^5^ A549 cells/ml densities (Scepter™ 2.0 Cell Counter, Merck) were transferred to a 96-well plate and incubated with series of five sample dilutions (D1:2, first dilution as half the equivalent of 1 mL of the sample) for 48 h at 5% CO2, 37°C, and humid atmosphere.

The 3-(4,5-dimethylthiazol-2-yl)-2,5-diphenyltetrazolium bromide (MTT) assay was used to determine cell viability, measured at 510 nm (ELISA LEDETECT 96, biomed Dr. Wieser GmbH; MikroWin 2013SC software), as previously described (29). The lowest concentration dropping absorption to <50% of cell metabolic activity (IC50) was defined as threshold toxicity level.

### 2.8 Statistical analysis

Data were analyzed in SPSS statistical software, version 27.0 for windows. The results were considered significant at the 5% significance level. To test the normality of the data, the Kolmogorov–Smirnov test or the Shapiro–Wilk test were used, according to the sample size. To characterize the sample, frequency analysis was used (n, %) for qualitative data and for quantitative data, the logarithm of bacterial and fungal counts and resistance to azoles was used. To compare bacterial and fungal contamination and resistance to azoles between two independent groups, the Mann–Whitney test was used (evaluate season effect, to compare industries in the summer, to compare the type of workplaces assessed), and between k > 2 independent groups (to compare industries in the autumn), the Kruskal-Wallis test was used, since the normality assumption was not verified. When statistically significant differences were detected, the Kruskal-Wallis, multiple comparison tests were used. To compare the culture media, the Wilcoxon Signed Ranks (comparison of two media) and Friedman (comparison of k > 2 media) tests were used, since the assumption of normality was not verified. To study the relationship between bacterial, fungal and resistance to azoles by sampling method, Spearman correlation coefficient was used. To assess species diversity, Simpson and Shannon indices, given by $\mathrm{Shannon Index}\;\left(H\right)=-\overset{s}{\underset{i=1}{\sum }}p_{i}\ln\left(p_{i}\right)$ and $\mathrm{Simpson Index}\;\left(D\right)=\frac{1}{\sum_{i=1}^{s}p_{i}^{2}}$ , were used, where pi is the proportion (ni/n) of individuals of one particular species found (ni) divided by the total number of individuals found (n).

## 3 Results

### 3.1 Viable bacterial contamination

**Figure 2:**
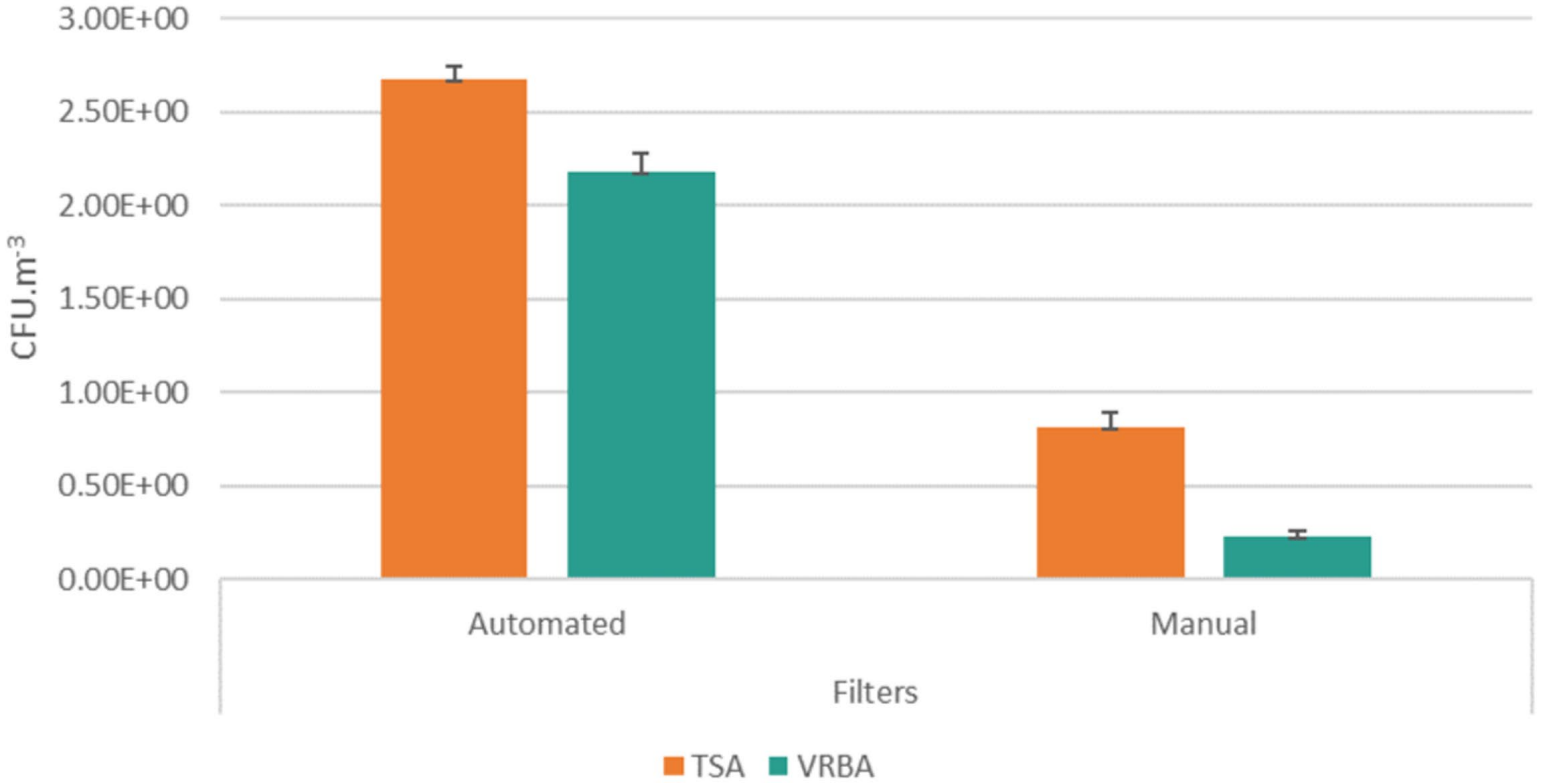
Bacterial distribution (total bacteria, TSA; Gram negative bacteria, VRBA) in filter samples from automated and manual industries (CFU.m − 3) and the standard error for each case.

Personal filter samples had the highest counts on total bacterial contamination (Manual: 8.15 × 10^1^ CFU.m^−3^; Automated: 2.67 × 10^5^ CFU.m^−3^), compared to the counts of Gram-negative bacteria (Manual: 2.29 × 10^1^ CFU.m^−3^; Automated: 2.18 × 10^2^ CFU.m^−3^) (Figure 2).

**Figure 3:**
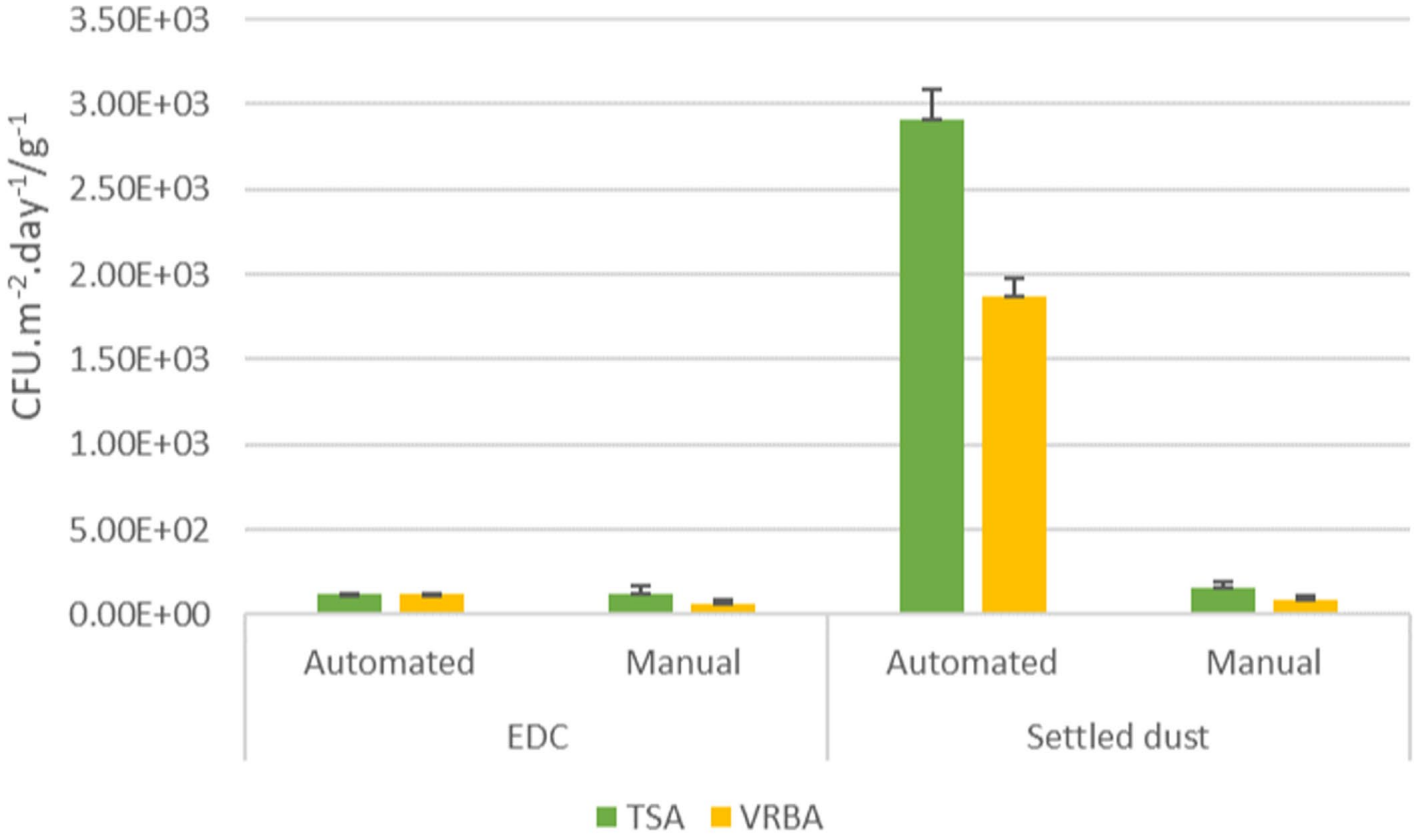
Bacterial distribution (TSA; VRBA) in automated and manual industries among the passive sampling matrices (EDC: CFU.m^−2^.day^−1^; Settled dust: CFU.g^−1^) and the standard error for each case.

EDC total bacterial counts ranged between 1.21 × 10^2^ CFU.m^−2^.day^−1^ in automated industries and 1.21 × 10^2^ CFU.m^−2^.day^−1^ in manual industries. As for Gram-negative counts, automated industries presented 1.21 × 10^2^ CFU.m^−2^.day^−1^, while on manual industries presented 6.07 × 10^1^ CFU.m^−2^.day^−1^. Total bacteria counts in settled dust ranged from 2.92 × 10^3^ CFU.g^−1^ in automated industries to 1.57 × 10^2^ CFU.g^−1^ in manual industries, whereas Gram-negative counts ranged from 1.81 × 10^3^ to 8.87 × 10^1^ CFU.g^−1^, respectively on automated and manual industries (Figure 3).

**Figure 4:**
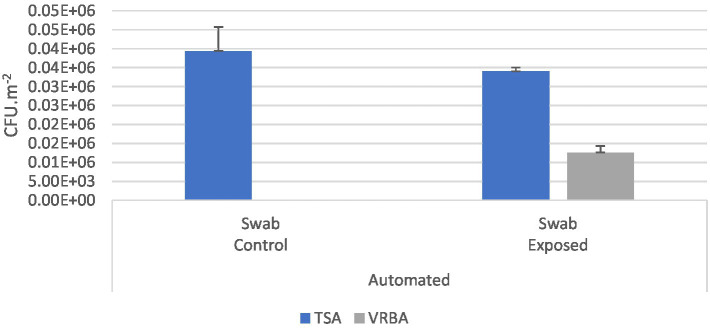
Bacterial (TSA; VRBA) distribution in automated industries in swabs from the workers’ hands (CFU.m^−2^) and the standard error for each case.

In swabs from workers’ hands, the contamination of total bacteria in control workers was 3.94 × 10^6^ CFU.m^−2^ while on exposed workers was 3.40 × 10^6^ CFU.m^−2^. Considering Gram-negative bacteria, no contamination was detected on control workers, while on exposed workers contamination reached 1.26 × 10^6^ CFU.m^−2^ (Figure 4).

### 3.2 Viable fungal contamination

**Figure 5:**
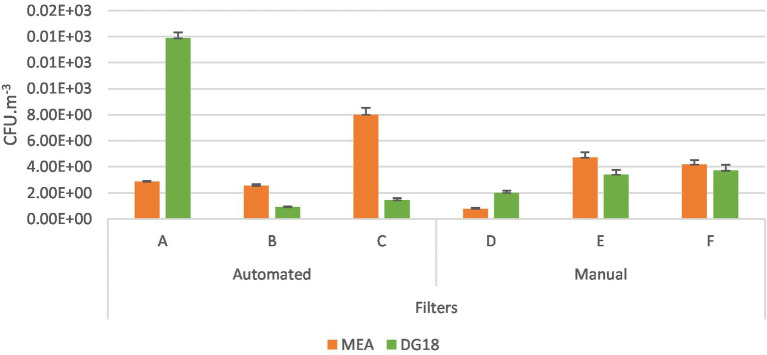
Fungal distribution (MEA; DG18) in automated and manual industries among filter samples (CFU.m^−3^) and the standard error for each case.

Personal filter samples had higher counts in company A (MEA 2.85 × 10^2^ CFU.m^−3^; DG18 1.39 × 10^3^ CFU.m^−3^) and C (MEA 8.0 × 10^2^ CFU.m^−3^; DG18 1.43 × 10^2^ CFU.m^−3^) among the automated industries. On the manual industries, industry E (MEA 4.7 × 10^2^ CFU.m^−3^; DG18 3.40 × 10^2^ CFU.m^−3^) and F (MEA 4.17 × 10^2^ CFU.m^−3^; DG18 3.70 × 10^2^ CFU.m^−3^) had the highest fungal counts (Figure 5).

EDC had the highest counts in industry A (DG18 7.58 × 10^0^ CFU.m^−2^.day^−1^) and D (MEA 7.58 × 10^1^ CFU.m^−2^.day-1; DG18 1.21 × 10^2^ CFU.m^−2^.day^−1^), while the settled dust samples, the counts ranged from 7.90 × 10^1^ CFU.g^−1^ in industry C to 3.59 × 10^2^ CFU.g^−1^ in industry A on DG18, and from1.84 × 10^2^ CFU.g^−1^ in industry A to 2.75 × 10^2^ CFU.g^−1^ in industry D on MEA (Supplementary Figures S1A,B).

**Figure 6:**
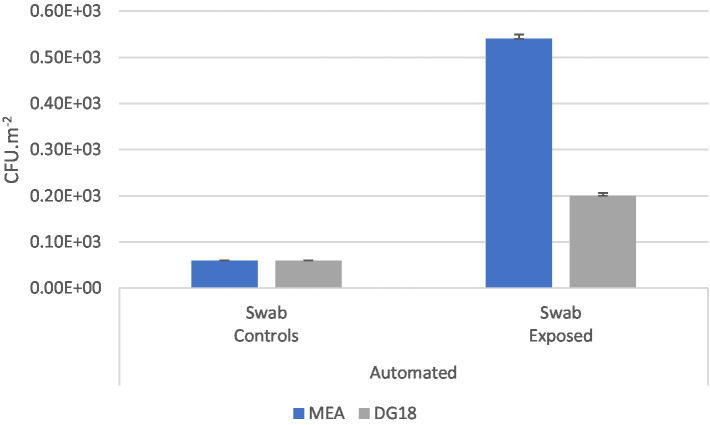
Fungal (MEA; DG18) counts in swabs from the hands of workers at the automated industries (CFU.m^−2^) and the standard error for each case.

Higher counts were observed in eSwabs from exposed workers (MEA 5.40 × 10^4^ CFU.m^−2^; DG18 2.00 × 10^4^ CFU.m^−2^) than in control workers (MEA 6.00 × 10^3^ CFU.m^−2^; DG18 6.00 × 10^3^ CFU.m^−2^) (Figure 6).

*Penicillium* sp. was the most prevalent fungal genus in personal airborne filter samples from both automated and manual industries (Table 2).

**Table 2:** Fungal distribution in industries A to F on filters (log [CFU.m^−3^]).

Matrix	Workplaces assessed plant	Industry	MEA			DG18		
			ID	CFU.m^−3^	%	ID	CFU.m^−3^	%
Filters	Automated	A	*Aspergillus* sp. *Penicillium* sp.	2.15 × 10^1^ 2.64 × 10^2^	7.6 92.4	*Aspergillus* sp. *Penicillium* sp.	2.17 × 10^1^ 1.36 × 10^3^	1.6 98.4
		B	*Cladosporium* sp. *Aspergillus* sp. *Penicillium* sp. *Rhizopus* sp.	1.37 × 10^0^ 4.00 × 10^1^ 1.36 × 10^2^ 7.59 × 10^1^	0.5 15.8 53.7 30.0	*Aspergillus* sp. *Penicillium* sp.	2.54 × 10^0^ 8.70 × 10^1^	2.8 97.2
		C	*Aspergillus* sp. *Penicillium* sp. *Rhizopus* sp.	6.91 × 10^1^ 5.47 × 10^2^ 1.84 × 10^2^	8.6 68.3 23.0	*Aspergillus* sp. *Cladosporium* sp. *Penicillium* sp.	3.85 × 10^1^ 1.14 × 10^0^ 1.4 × 10^2^	26.9 0.8 72.3
	Manual	D	*Cladosporium* sp. *Aspergillus* sp. *Penicillium* sp. *Rhizopus* sp.	2.11 × 10^0^ 3.17 × 10^0^ 6.97 × 10^1^ 3.16 × 10^0^	2.7 4.1 89.2 4.0	*Mucor* sp. *Aspergillus* sp. *Penicillium* sp.	5.29 × 10^0^ 1.08 × 10^0^ 1.94 × 10^2^	2.6 0.5 96.8
		E	*Aspergillus* sp. *Penicillium* sp. *Rhizopus* sp.	4.44 × 10^0^ 4.48 × 10^2^ 1.78 × 10^1^	0.9 95.3 3.8	*Aspergillus* sp. *Penicillium* sp. *Syncephalastrum racemosum*	6.67 × 10^0^ 3.31 × 10^2^ 2.22 × 10^0^	2.0 97.4 0.7
		F	*Aspergillus* sp. *Paecilomyces* sp. *Penicillium* sp.	2.79 × 10^1^ 1.13 × 10^0^ 3.88 × 10^2^	6.7 0.3 93.0	*Aspergillus* sp. *Penicillium* sp.	1.23 × 10^1^ 3.58 × 10^2^	3.3 96.7

Regarding automated plants, *Penicillium* sp. was the most prevent genus in industries A (EDC: 100% DG18; SD: 64.1% MEA, 77.7% DG18), B (SD: 71.3% MEA, 83.5% DG18) and C (SD: 83.1% MEA, 65.8% DG18). In the manual industries, *Penicillium* sp. was the most prevalent genus in industry D (EDC: 60% MEA, 100% DG18; SD: 82.5% MEA, 82.5% DG18) (Supplementary Table S1). *Penicillium* sp. was also the most prevalent genus in eSwab samples at automated plants, except unexposed controls from industry A where the most prevalent genus was *Cladosporium* sp. (66.7% MEA) (Supplementary Table S2).

Among *Aspergillus* sections present in MEA, *Nigri* was the most prevalent section in personal filter samples from workers at industries A, B, C, D and E (100%). *Fumigati* section also showed to be prevalent in filter samples (Industry F 67.8%).

**Figure 7:**
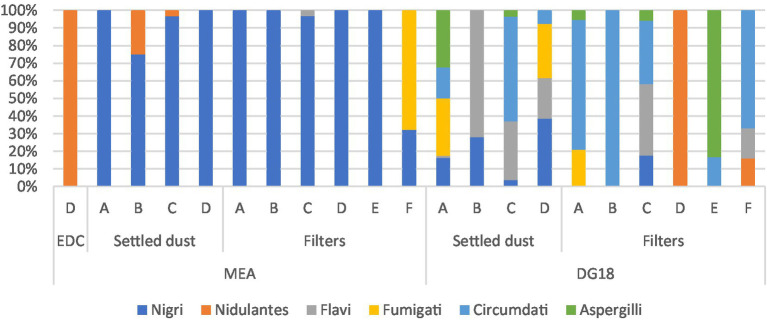
*Aspergillus* sections distribution in DG18 and MEA in industries A to F (Filters: CFU.m^−3^; EDC: CFU.m^−2^.day^−1^; Settled dust: CFU.g^−1^).

The most prevalent *Aspergillus* section in filters on DG18 were *Circumdati* (Industries A 73.7%; B 100%; C 36%; E 16.7%; F 67%), *Nidulantes* and *Aspergilli* (industry D 100%; E 83.3%, respectively). The most prevalent *Aspergillus* section in EDC samples cultivated on MEA was *Nidulantes* (100%), while in settled dust samples, *Nigri* was the most prevalent section in industries A (100%), B (75%), C (96.7%), and D (100%). The second most prevalent section in MEA was *Nidulantes* in settled dust (Industry B 25%; D 3.33%). When cultivating on DG18, the most prevalent section was *Flavi* (A: 1.3%; B: 72%; C: 3.7%; and D: 23.1%). The second most prevalent sections were *Nigri* (A: 16.3%; B: 28%; C: 33.3%; and D 38.5%) and *Circumdati* (A: 17.5%; C: 59.3%; D: 7.7%) (Figure 7).

In eSwabs from exposed workers’ hands, either in MEA and DG18, the sections *Nigri* and *Fumigati* were the only *Aspergillus* sections identified (Plant B 100%).

### 3.3 Fungal distribution in azole-supplemented media

**Figure 8:**
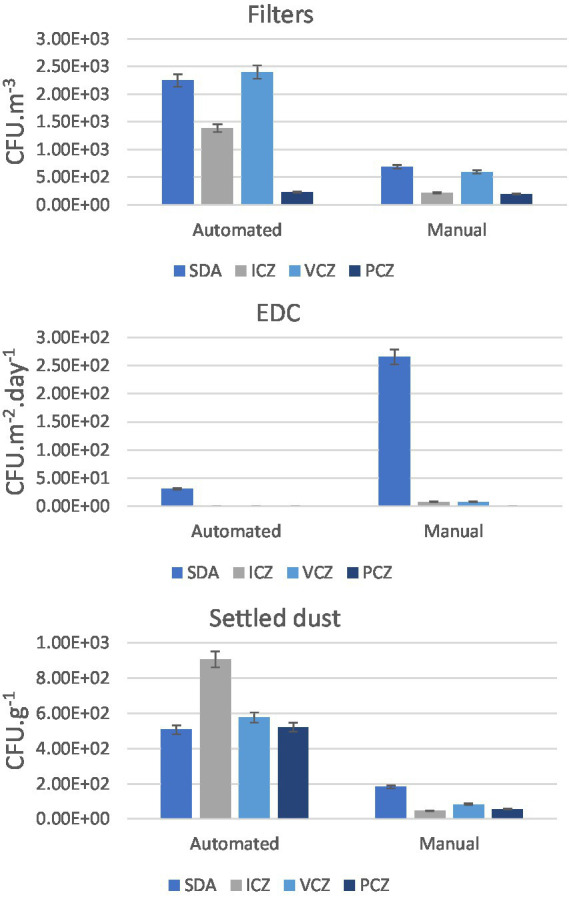
Fungal counts in azole screening in automated and manual industries, per sampling method: Filters (CFU.m^−3^); EDC (CFU.m^−2^.day^−1^); Settled dust (CFU.g^−1^) and the standard error for each case.

The burden of fungal resistance is depicted in Figure 8 per industry type (automated vs. manual). Higher fungal counts with reduced azole susceptibility were observed in the automated industry by filter and settled dust sampling.

Regarding fungal diversity in azole-supplemented media (Table 3), *Penicillium* sp. was the most prevalent (ICZ, VCZ and PCZ) by filter sampling, followed by *Mucorales* order (*Mucor* sp., *Rhizopus* sp.) (PCZ and ICZ). *Aspergillus* sp. was also present in filters from manual (ICZ and PCZ) and automated (VCZ and PCZ) plants. Five *Aspergillus* sections were identified, including *Circumdati* (VCZ and ICZ) and *Nidulantes* (PCZ) with reduced susceptibility to azoles.

**Table 3:** Fungal diversity in azole screening per industry type.

			SDA	4 mg/L ICZ	2 mg/L VCZ	0.5 mg/L PCZ
Matrix	Plant type	ID	CFU.m^−3^	CFU.m^−3^	CFU.m^−3^	CFU.m^−3^
Filters	Manual	*Alternaria* sp.		6.41E+00		
		*Aspergillus* sp.	4.29E+02	4.90E+00	1.19E+00	1.21E+01
		*Chrysosporium* sp.		4.75E+00	2.05E+01	1.28E+01
		*Cladosporium* sp.		7.61E+00	1.85E+02	5.10E+01
		*Fusarium verticilloides*				1.44E+00
		*Fusarium solani*				1.19E+00
		*Lichtheimia* sp.			2.24E+00	
		*Mucor* sp.	5.80E+01	2.80E+01	9.45E+01	1.34E+01
		*Paecilomyces* sp.			2.22E+01	1.30E+00
		*Penicillium* sp.	2.38E+03	1.48E+03	2.59E+03	4.48E+02
		*Rhizopus* sp.	3.69E+01	6.06E+01	7.92E+01	3.71E+00
		*S. racemosum*	3.21E+01	2.22E+00	7.07E+00	
	Automated	*Alternaria* sp.		6.41E+00		
		*Aspergillus* sp.	4.14E+02		1.19E+00	1.21E+01
		*Chrysosporium* sp.			2.05E+01	1.28E+01
		*Cladosporium* sp.		2.33E+00	1.85E+02	5.10E+01
		*F. verticilloides*				1.44E+00
		*Fusarium solani*				1.19E+00
		*Lichtheimia* sp.			2.24E+00	
		*Mucor* sp.	5.69E+01	2.69E+01	9.45E+01	1.34E+01
		*Paecilomyces* sp.			2.22E+01	1.30E+00
		*Penicillium* sp.	1.74E+03	1.30E+03	2.59E+03	4.48E+02
		*Rhizopus* sp.	3.47E+01	5.19E+01	7.92E+01	3.71E+00
		*S. racemosum*			7.07E+00	

Filters: [CFU.m^−3^].

Settled dust and EDC sampling also enabled the identification of *Mucor* sp. and *Rhizopus* sp. in azole-supplemented media, but not *Aspergillus* sp. (Supplementary Table S3).

### 3.4 Detection of the targeted fungal sections

Regarding the four *Aspergillus* sections investigated by PCR, two of them were detected. *Aspergillus* section *Fumigati*, was detected in settled dust samples (4 out of 29, 13.79%) and in filter samples (1 out of 58, 1.72%). *Aspergillus* section *Circumdati* was detected in settled dust samples (1 out of 29, 3.45%), and also in filter samples (2 out of 58, 3.45%) (Supplementary Table S4).

### 3.5 Cytotoxicity results

Based on the ability to decrease cell metabolic activity (IC50), cytotoxicity levels were determined in extracts of personal filters and settled dust samples as depicted in Table 4. Six percent of the filter sample extracts were highly cytotoxic for A549 cells, while most filters were low cytotoxic for both cell lines. Regarding settled dust, 18% were highly cytotoxic for A549 cells, and 45% were highly cytotoxic for HepG2 cells.

**Table 4:** Cytotoxicity levels of filters (*N* = 18) and settled dust (*N* = 11) diluted samples in A549 and HepG2 cellular lines.

	A549 cells				HepG2 cells			
Cytotoxicity level	High	Moderate	Low	n.d.	High	Moderate	Low	n.d.
Filters (N)	1	0	14	3	0	4	12	2
Settled dust (N)	2	4	3	2	5	2	1	3

Cytotoxicity level: High, IC50 at third or more dilutions; Moderate, IC50 at second dilution; Low, IC50 at first dilution; n.d., no cytotoxicity detected.

### 3.6 Comparisons and correlation analysis

Between summer and autumn, statistically significant differences were detected among filter samples regarding: (i) bacterial counts in TSA (*p* = 0.006) and VRBA (*p* < 0.0001) with statistically highest bacterial counts during summer; (ii) fungal counts in MEA (*p* = 0.001), with statistically highest fungal counts during autumn. Statistically significant differences were also detected in azole screening in ICZ, VCZ and PCZ (*p*’s < 0.05) with highest values during summer. In settled dust samples, statistically significant differences were detected between summer and autumn regarding to: (i) bacterial counts in TSA (*p* < 0.05) and VRBA (*p* < 0.05) with statistically highest bacterial counts during summer; (ii) azole screening in ICZ, VCZ and PCZ (*p*’s < 0.05) with highest values during summer. In the eSwabs samples, statistically significant differences were detected in relation to bacterial counts in TSA (*p* = 0.001), with, once again, highest bacterial counts during summer (Supplementary Table S5).

As statistically significant seasonal variation was identified, the following analyzes were carried out separately by season. During summer in the filters, statistically significant differences were detected between the industries A and B, concerning bacterial counts in TSA (*p* = 0.005) and VRBA (*p* = 0.035), with industry B revealing higher counts and relatively to azole screening in ICZ (*p* = 0.029), with industry A revealing higher counts. In settled dust and swabs, no statistically significant differences were detected between industries A and B (*p*’s > 0.05) (Supplementary Table S6).

Statistically significant differences were detected in autumn measurements among industries in the filters, regarding to: (i) bacterial counts in TSA (*p* = 0.025), with industry B having the highest bacterial counts and industries A and D with lower counts, and in VRBA (*p* = 0.002), with industry D having the highest bacterial counts, while industries B, C, E and F with lower counts; (ii) fungal counts in MEA (*p* = 0.002), with industries C and E showing the highest fungal counts; (iii) fungal counts in SDA (*p* = 0.001), with industry E showing higher counts followed by industry C, in VCZ (*p* = 0.002), with industry D showing higher counts followed by industry B and in PCZ (*p* < 0.0001), with industry D revealing the highest values (Supplementary Table S7). As for *Aspergillus* sp., no statistically significant differences were detected (*p* > 0.05). Concerning settled dust sample method, statistically significant differences were detected regarding to: (i) bacterial counts in TSA (*p* = 0.011) and in VRBA (*p* = 0.024), with industry A revealing the highest counts; (ii) fungal counts in SDA (*p* = 0.008), with industry C showing higher values, followed by industry D, and in PCZ (*p* = 0.018), with industry A revealing the highest values, followed by industry D (Supplementary Table S7). With respect to eSwabs, no statistically significant differences were detected between industries (*p*’s > 0.05).

The comparison of bacterial counts (TSA and VRBA), fungal counts (MEA and DG18) and azole screening (SDA, ICZ, VCZ and PCZ) in the two types of industries assessed (automated/manual) was only possible during autumn, as only automated industries were assessed during summer. In filters, statistically significant differences between manual and automated industries were only detected for fungal counts in PCZ (*p* = 0.047), with manual industries having higher values. In settled dust, no statistically significant differences were detected (Supplementary Table S8). This analysis could not be performed on eSwabs, as data were not collected for the manual workplaces. It was also not possible to perform for the EDC, since there were only two observations. Considering *Aspergillus* sp., no statistically significant differences were detected between the types of industry (*p* > 0.05) (Supplementary Table S1).

Statistically significant differences in bacterial counts were detected between TSA and VRBA in filter samples (*p* = 0.020), with the VRBA medium presenting lower counts. Regarding fungal counts, no statistically significant differences were detected between MEA and DG18 (*p* = 0.943). Regarding azole screening, statistically significant differences were detected between culture medium (*p* < 0.0001). In Friedman’s paired multiple comparisons the differences were between the PCZ and the other media (*p*’s < 0.05) with PCZ having the lowest values (Supplementary Table S9).

In settled dust, bacterial counts in VRBA were also statistically significant lower than counts in TSA (*p* < 0.0001). No statistically significant differences were detected among fungal counts in MEA and DG18 (*p* = 0.873) nor among azole-supplemented media (Supplementary Table S9).

In eSwabs from the exposed workers, bacterial counts in VRBA were statistically significant lower than bacterial counts in TSA (*p* < 0.0001), whereas fungal counts in DG18 were statistically significant lower than fungal counts in MEA (*p* = 0.013) (Supplementary Table S9).

The azole screening and the EDC sampling method were excluded from this analysis, as the observations number was very small.

Concerning the sampling method (particularly, filters in TSA), a relation between higher bacterial counts in filters in TSA and higher values in SDA was determined. Higher bacterial counts in VRBA was related to lower fungal counts in MEA and higher fungal counts in VCZ and PCZ. Higher fungal counts in MEA was related to higher counts in DG18 and SDA and lower values in VCZ and PCZ. Higher fungal counts in DG18 was related to higher values in SDA. In azole screening, higher fungal counts in a given culture medium were related to higher values in another (Supplementary material Text S1; Table 5).

**Table 5:** Study of the relationship between bacterial, fungal and resistance to azoles counts by sampling method.

	Culture media	Filter						
		Bacteria	Fungi		Azole screening			
		VRBA	MEA	DG18	SDA	ICZ	VCZ	PSZ
Bacteria	TSA	0.172	0.040	−0.150	0.374^*^	−0.007	0.149	0.034
	VRBA		−0.563^**^	−0.126	−0.174	0.233	0.448^**^	0.528^**^
Fungi	MEA			0.373^**^	0.582^**^	−0.116	−0.304^*^	−0.483^**^
	DG18				0.382^**^	0.113^*^	−0.091	−0.159
Azole screening	SDA					0.088	0.119	−0.227
	ICZ						0.745^**^	0.648^**^
	VCZ							0.726^**^
Settled dust								
Bacteria	TSA	0.979^**^	−0.470^*^	0.310	0.295	0.918^**^	0.791^**^	0.807^**^
	VRBA		−0.487^**^	0.289	0.306	0.932^**^	0.764^**^	0.799^**^
Fungi	MEA			0.281	0.193	−0.404^*^	−0.431^*^	−0.625^**^
	DG18				0.304	0.299	0.132	0.174
Azole screening	SDA					0.172	0.053	0.071
	ICZ						0.793^**^	0.784^**^
	VCZ							0.658^**^
Swabs								
Bacteria	TSA	0.332^*^	0.152	0.094				
	VRBA		0.032	−0.131				
Fungi	MEA			0.171				
	DG18							
Azole screening	SDA							
	ICZ							
	VCZ							

Spearman correlation results.

** Correlation is significant at the 0.01 level (2-tailed).

* Correlation is significant at the 0.05 level (2-tailed).

In settled dust, higher bacterial counts in TSA were related with higher counts in VRBA, lower counts on MEA and higher counts in ICZ, VCZ and PCZ. Higher bacterial counts in VRBA were related with lower fungal counts in MEA and higher values in ICZ, VCZ and PCZ. Higher fungal counts in MEA were related with lower values in ICZ, VCZ and PCZ. Higher values in ICZ were related with higher values in VCZ and PCZ, and higher values in VCZ were related with higher values in PCZ (Supplementary material Text S1; Table 5).

Considering the surfaces eSwabs sampling method, a significant correlation with weak intensity suggested that higher bacterial counts in TSA is related with higher counts in VRBA (Supplementary material Text S1; Table 5).

The highest fungal diversity was found in DG18 inoculated with the eSwabs of the exposed workers from automated industry B (Shannon Index (H) = 1.34 and Simpson Index (D) = 2.95), followed by filters also from industry B inoculated in MEA (Shannon Index (H) = 1.01 and Simpson Index (D) = 2.48) (Supplementary Table S10).

## 4 Discussion

Occupational exposure to microorganisms during waste handling is a known health hazard for exposed workers (9, 30–31). Although microbial composition of bioaerosols in traditional waste sorting has been described previously (18, 32, 33) the work environment microbiome is rarely described at automated waste sorting plants (19, 21).

The present study compares (by personal air sampling and passive methods) workers’ exposure to microbial agents in waste sorting in modern automated facilities with exposure in traditional facilities, addressing selected pathogens and fungal resistance. The use of complementary sampling methods (personal, environment) and laboratorial assays (culture-based identification, molecular detection, *in vitro* cytotoxicity) allow to identify a wider spectrum of the microbiota, and screen for potential health risks in this occupational setting (18).

Despite the restricted number of assessed plants, this study confirmed a high exposure to microbial agents. The use of six selected fungal molecular targets in this study allowed comparison with previous molecular results (19, 21). The selection of these molecular targets, specific to the environment under study, was based on results from previous studies that described fungal contaminants with clinical and toxicological relevance (2, 18). The toxicological assessment of microbes is frequently done by *in vitro* assays. Previous studies in these environments indicated that dust samples and personal air samples contained ligands capable of stimulating TLR2 and TLR4 receptors, with the potential to evoke an inflammatory response in exposed workers (9, 20). In this study, the MTT assay was used to assess cell viability of A549 and HepG2 cells after exposure to dust and personal air samples.

### 4.1 Compliance assessment

In personal filter samples the guidelines for total bacteria (10,000 CFU.m^−3^) were not overpassed in either automated or manual industries (34, 35), as well as in the case of gram-negative bacteria (1,000 CFU.m^−3^) (34). Concerning fungi, one automated industry (A) surpassed the guidelines (1,000 CFU.m^−3^) (34, 36) and, although with lower counts than other studies performed in the same setting (18, 37, 38), this fact claim attention for the need of intervention in the scope of microbial agents’ risk management, even in automated industries, with less workers engaged in the different tasks. Thus, probably other variables that were not studied influence the contamination and not the type of process (manual or automatic).

### 4.2 Sampling and analyses approaches

For a better estimation of workers’ health risks in waste sorting industries, a comprehensive sampling strategy using complementary sampling methods is of the upmost importance. An important feature of this study is the evaluation of the viable microbiota, due to the critical implication of microorganisms’ viability in the health effects that can be observed, thus, being a more useful resource for accurate risk assessments (39). The use of previously described methods also enables the generation of comparable data among different studies (2, 18). Besides, we should be aware of the drawbacks to apply only molecular tools when assessing occupational exposure to microbial contamination. In fact, despite cultivation of microorganisms induce a bias in their representation (40–42) we cannot neglect the fact that the isolation of fungal isolates is vital to understand and study specific isolates (such as the ones presenting azole resistance) and to better characterize the biodiversity present in a specific occupational environment (41). Nevertheless, in automated plants EDC for sampling were used in the control room/office area of the respective plants (the expected “cleaner” areas from the facilities) and no contamination was observed, corroborating the suitability of the sampling approach.

The surveillance of antifungal resistance is considered to be critical in hot spot environments such as waste management, due to the foreseen increased prevalence of resistant fungi as an indirect consequence of climate change (2, 3, 18, 43). Indeed, previous detection of azole-resistant *Aspergillus fumigatus* harboring the TR34/L98H Mutation in a waste management facility justifies this (10). Thus, the application of multiple culture conditions (combining different culture media and incubation temperatures), used in parallel with more refined molecular methods, will provide complementary information regarding microbial diversity and, in particular, fungal diversity (41, 44). All these datasets will provide information to characterize in detail exposure and estimate all the possible impacts on workers’ health (2, 41).

### 4.3 Fungal contamination and azole resistance screening

The seasonal influence on viable microbial contamination observed in this study, including on fungi with reduced susceptibility to the tested azoles, raises concern on the impact of climate change on the development of antimicrobial resistance (AMR). It is well described that the continuing disturbance of the environment, with extreme weather events and higher global temperatures, impacts the emergence and spread of antimicrobial resistance phenotypes in the environment (43, 45, 46). In this context, specific fungal species are expected to thrive through climate change, boosting crops’ contamination by toxigenic fungal species with consequent increase of the use of fungicides. Thus, not only environmental pressures may result in new fungal diseases (47), they can also increase human exposure to mycotoxins, and prompt the development of acquired azole resistance that hampers the management of life-threatening fungal invasive infections (43).

Driven by this real menace, the World Health Organization (WHO) recently published the first fungal priority pathogens list, identifying 19 groups of human fungal pathogens associated with a higher risk of mortality or morbidity (25). However, the concern regarding the toxigenic potential of specific fungal species, sections and strains was overlooked in the published WHO list, hindering a more accurate intervention concerning risk management measures. In fact, *Aspergillus* section *Flavi*, found in settled dust and filters samples, was not listed by WHO, although in previous studies performed in the waste sector the presence of this section resulted in occupational exposure to aflatoxin B1, a carcinogenic mycotoxin (7, 48). Furthermore, the section *Circumdati* (observed and detected by molecular tools in the same matrices), was also neglected in WHO list, although species from this section produce large amounts of ochratoxin A (OTA) (49). Several studies have linked OTA exposure with different human diseases, such as Balkan endemic nephropathy (BEN) and chronic interstitial nephropathy (CIN), as well as other renal diseases (50).

In this study, *Aspergillus* section *Fumigati,* that was listed as of critical priority by WHO and suggested as indicator of harmful fungal contamination in waste management industry (2, 18) was observed in filters and settled dust samples and detected by molecular tools in different settled dust samples, proving the widespread of this section in the assessed plants. *Fusarium* species (*F. solani* and *F. verticilloides*) and Mucorales (*Mucor*, *Rhizopus*, *Syncephalastrum* and *Lichtheimia* genera) (listed as of high priority by WHO) were also identified. In addition all the *Aspergillus* sections identified have toxigenic potential and this should be also considered when performing risk characterization.

The statistically significant lower fungal prevalence in posaconazole is in accordance with the reported superior activity of this azole (compared to itraconazole or voriconazole) against *Aspergillus* and *Mucormycetes* isolates (51). Nevertheless, the observed reduced susceptibility of pathogens of critical priority (Mucorales and *Fusarium* sp.) to posaconazole supports the need to intervene in this occupational environment. In filters, *Mucor* sp. and *Fusarium* sp. were observed in all azoles and in posaconazole only, respectively, with no differences between manual and automated industries; in settled dust, *Mucor* sp. prevalence in azoles was about 1.6-fold higher in the automated industries. Although no conclusions can be drawn regarding azole resistance as the tested azole concentrations are cut off values defined only for *Aspergillus* section *Fumigati* (not *Fusarium* sp. or Mucorales), these preliminary results raise awareness for the need of implementing surveillance programs dedicated to the fungal prioritized species in the environment.

### 4.4 Skin-biota samples

Strict hygiene regimes were in place, due to the ongoing pandemic, and many workers had sanitized their hands before eSwabs samples could be collected. However, the results report microbial contamination in both controls and exposed hands claiming attention for the possible exposure by hand to face/mouth contact even when strict hygienic measures are in place. The findings corroborate previous results concerning the prevalence of gastrointestinal symptoms, besides respiratory disorders, among the workers from the same units (19, 20).

### 4.5 Cytotoxic analyses

Cytotoxicity is one of the most important and preliminary indicators in biological risk assessment and *in vitro* toxicology (52). While chemical pollutants have been more studied through these type of resources, we propose an increment on the use of *in vitro* testing when performing environmental assessments to estimate biological effects resulting from exposure to biological agents. In this study, lower cell viability was observed for A549 and HepG2 cells exposed to settled dust, compared to cells exposed to filters. The analysis of the microbial counts in automated industries of filters and settled dust revealed a higher bacteria contamination in settled dust (2.92 × 10^3^ CFU.g^−1^ TSA and 1.87 × 10^3^ CFU.g^−1^ VRBA), and higher fungal counts in filters (8 × 10^2^ CFU.m^−3^ MEA and 1.39 × 10^3^ CFU.m^−3^ DG18). The lower cell viability observed with settled dust might be partially explained by their relatively high bacterial contamination or the prevalence of specific fungal species, besides other contaminants, such as mycotoxins, particles, or chemicals (not assessed in this study). Some phenomena well described are the cellular toxicity of toxigenic *Fusarium* sp. and its mycotoxins fumonisins (53), and *Aspergillus* section *Nidulantes* (series *Versicolores*) due to the production of sterigmatocystin with renal and hepatic toxicity (54). Not only these two fungal genus/species are potentially toxigenic and related to cytotoxicity *in vitro*, they were also found in filters with reduced susceptibility to posaconazole in this study. These findings also reinforce the need of surveillance of antifungal resistance in the environment for fungal priority species, as a contribution to proper antifungal stewardship from the environment to the bench.

## 5 Conclusion

This study allowed to conclude once again that working in manual and automated waste sorting plants imply high exposure to microbial agents. The approach followed, that comprehends several sampling methods and assays employed, is increasingly applied and industrial hygienists/exposure assessors should rely on this new trend to achieve a precise assessment of microbial risk.

It was possible to conclude that the fact of being automated does not result in a reduction in workers exposure to fungal pathogens associated with a high risk of mortality or morbidity. Moreover, the seasonal influence on viable microbial contamination observed claims attention for the potential impact of climate change in the occupational environment contamination and workers exposure pattern and, consequently, in the resulting health effects. Some findings should be highlighted: (a) one automated industry surpassed the guidelines for fungi (b) the presence of indicators of harmful fungal contamination (*Aspergillus* section *Fumigati*); (c) the identification of *Aspergillus* sections with toxigenic potential; (d) microbial contamination in both controls and exposed workers’ hands potentiating the exposure by hand to face/mouth contact; (e) the observed reduced azole susceptibility of pathogens of critical priority (Mucorales and *Fusarium* sp.).

*In vitro* tools are important tools to estimate the health effects related to the overall contamination present in the workplace environment. However, more efforts in science and engineering need to be developed to design and implement risk management measures more effective in controlling workers exposure in this occupational setting. This is of particular relevance due to the boost expected and already happening in the number of waste management plants across the European Union promoted by the needed circular economy goals.

## Data availability statement

The original contributions presented in the study are included in the article/Supplementary material, further inquiries can be directed to the corresponding author.

## Ethics statement

The studies involving humans were approved by the Regional Committees for Medical research Ethics South East Norway, REK South East (ref. no. 34312). The studies were conducted in accordance with the local legislation and institutional requirements. The participants provided their written informed consent to participate in this study. Ethical approval was not required for the studies on animals in accordance with the local legislation and institutional requirements because only commercially available established cell lines were used.

## Author contributions

CV: Conceptualization, Formal analysis, Funding acquisition, Investigation, Methodology, Project administration, Resources, Supervision, Writing – original draft, Writing – review & editing. EE: Formal analysis, Methodology, Validation, Writing – original draft. BG: Formal analysis, Writing – original draft. MD: Formal analysis, Writing – original draft. RC: Formal analysis, Writing – original draft. PP: Formal analysis, Writing – original draft. EC: Formal analysis, Writing – original draft. MT: Formal analysis, Funding acquisition, Validation, Writing – original draft. LC: Formal analysis, Writing – original draft. SV: Methodology, Writing – review & editing. PG: Funding acquisition, Writing – review & editing. AA: Writing – review & editing. AS: Funding acquisition, Methodology, Project administration, Resources, Supervision, Writing – review & editing.
