# Supplementary material for: Comprehensive assessment of occupational exposure to microbial contamination in waste sorting facilities from Norway
Source: Front Public Health. 2023 Dec 19;11:1297725. doi: 10.3389/fpubh.2023.1297725 (PMC10766354; doi:10.3389/fpubh.2023.1297725)
Supplement: Supplementary file 1 [file Data_Sheet_1.docx]

Supplementary Material

# Supplementary Figures and Tables

## Supplementary Figures


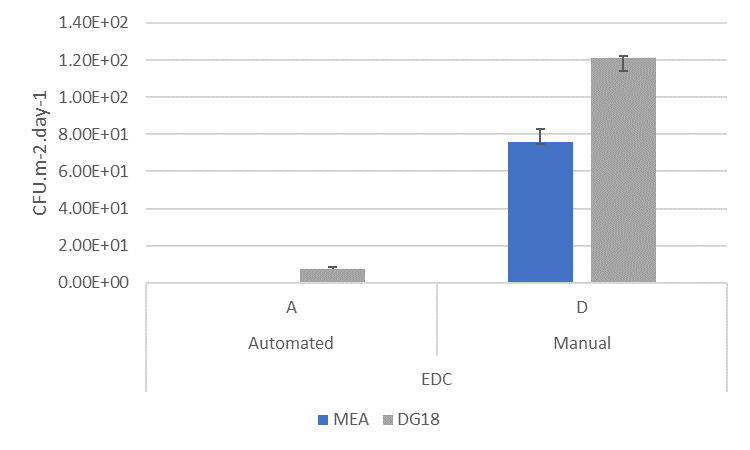


Figure S1 A - Fungal distribution (MEA; DG18) in automated and manual industries among passive sampling methods (EDC: CFU.m^−2^.day^−1^) and the standard error, for each case.


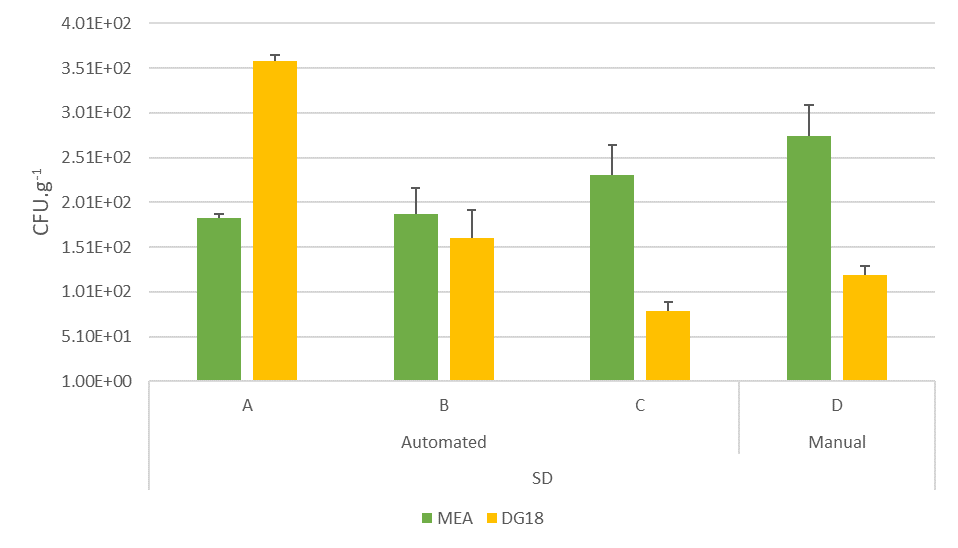


Figure S1 B - Fungal distribution (MEA; DG18) in automated and manual industries among passive sampling methods (Settled dust: C FU.g^−1^) and the standard error, for each case.

## Supplementary tables

Table S1 - Fungal distribution in industries A to F on passive sampling methods (CFU.m^−2^.day^−1^/CFU.g^−1^).

| **Type of industry assessed** | **Industry** | **Matrix** | **MEA** | | | **DG18** | | |
| --- | --- | --- | --- | --- | --- | --- | --- | --- |
|  |  |  | **ID** | **CFU.m^−2^.day^−1^/CFU.g^−1^** | **%** | **ID** | **CFU.m^−2^.day^−1^/CFU.g^−1^** | **%** |
| **Automated** | **A** | EDC |  | | | *Penicillium* sp. | 7.58 x 10^0^ | 100 |
|  |  | Settled dust | *Aspergillus* sp.  *Rhizopus* sp.  *Penicillium* sp.  *Mucor* sp. | 4.30 x 10^1^  1.00 x 10^1^  1.18 x 10^2^  1.30 x 10^1^ | 23.4  5.4  64.1  7.1 | *Penicillium* sp.  *Aspergillus* sp. | 2.05E+02  8.0 x 10^1^ | 77.7  22.3 |
|  | **B** | Settled dust | *Aspergillus* sp.  *Penicillium* sp.  *Rhizopus* sp.  *Syncephalastrum racemosum* | 3.60 x 10^1^  1.34 x 10^2^  9.0 x 10^0^  9.0 x 10^0^ | 19.2  71.3  4.8  4.8 | *Aspergillus* sp.  *Penicillium* sp.  *Cladosporium* sp. | 2.5 x 10^1^  1.32 x 10^2^  1.0 x 10^0^ | 15.8  83.5  0.6 |
|  | **C** | Settled dust | *Aspergillus* sp.  *Penicillium* sp.  *Rhizopus* sp. | 3.0 x 10^1^  1.92 x 10^2^  9.0 x 10^0^ | 13.0  83.1  3.9 | *Aspergillus* sp.  *Penicillium* sp. | 2.70 x 10^1^  5.20 x 10^1^ | 34.2  65.8 |
| **Manual** | **D** | EDC | *Aspergillus* sp.  *Penicillium* sp. | 3.03 x 10^1^  4.55 x 10^1^ | 40  60 | *Penicillium* sp. | 1.21 x 10^2^ | 100 |
|  |  | Settled dust | *Aspergillus* sp.  *Paecilomyces* sp.  *Penicillium* sp.  *Rhizopus* sp. | 2.70 x 10^1^  1.10 x 10^1^  2.27 x 10^2^  1.00 x 10^1^ | 9.8  4.0  82.5  3.6 | *Aspergillus* sp.  *Cladosporium* sp.  *Penicillium* sp. | 1.30 x 10^1^  8.00 x 10^0^  9.90 x 10^1^ | 10.8  6.7  82.5 |

Table S2 - Fungal distribution in eSwabs from workers´ hands in industries A, B and C (CFU.m^-2^).

| **Workplaces assessed** | **Matrix** | **Industry** | **MEA** | | | **DG18** | | |
| --- | --- | --- | --- | --- | --- | --- | --- | --- |
|  |  |  | **ID** | **CFU.m^-2^** | **%** | **ID** | **CFU.m^-2^** | **%** |
| **Automated** | Swabs Controls | **A** | *Penicillium* sp.  *Cladosporium* sp. | 2.0 x 10^3^  4.0 x 10^3^ | 33.3  66.7 |  |  |  |
|  |  | **C** |  |  |  | *Penicillium* sp. | 6.0 x 10^4^ | 100 |
|  | Swabs Exposed | **A** | *Penicillium* sp. | 1.0 x 10^4^ | 100 | *Cladosporium* sp.  *Penicillium* sp. | 2.0 x 10^3^  6.0 x 10^3^ | 25  75 |
|  |  | **B** | *Alternaria* sp.  *C. sitophila*  *Cladosporium* sp.  *Aspergillus* sp.  *Penicillium* sp.  *Phoma* sp. | 2.0 x 10^3^  2.0 x 10^3^  1.20 x 10^4^  4.0 x 10^3^  2.20 x 10^4^  2.0 x 10^3^ | 4.5  4.5  27.3  9.1  50  4.5 | *Chrysosporium* sp.  *Aspergillus* sp.  *Penicillium* sp. | 2.0 x 10^3^  2.0 x 10^3^  8.0 x 10^3^ | 16.7  16.7  66.7 |

Table S3 - Fungal diversity in azole screening per industry type. EDC: [CFU.m^−2^.day^−1^]. Settled dust: [CFU.g^−1^].

| **Matrix** | **Industry type** | **ID** | **SDA** | **%** | **4 mg/L ICZ** | **%** | **2 mg/L VCZ** | **%** | **0.5 mg/L PCZ** | **%** |
| --- | --- | --- | --- | --- | --- | --- | --- | --- | --- | --- |
| EDC | Automated | *Penicillium* sp. | 3.03E+01 | 100% |  |  |  |  |  |  |
|  | Manual | *A.* section *Circumdati* | 7.58E+00 | 4% |  |  |  |  |  |  |
|  |  | *Chrysosporium* sp. |  |  |  |  | 7.58E+00 | 100% |  |  |
|  |  | *Penicillium* sp. | 1.97E+02 | 96% |  |  |  |  |  |  |
| settled dust | Automated | *A.* section *Flavi* | 4.00E+00 | 1% |  |  |  |  |  |  |
|  |  | *A.* section *Nigri* | 1.50E+01 | 3% |  |  |  |  |  |  |
|  |  | *Cladosporium* sp. |  |  | 2.07E+02 | 23% |  |  | 3.08E+02 | 59% |
|  |  | *Mucor* sp. | 1.05E+02 | 19% | 3.22E+02 | 36% | 9.30E+01 | 16% | 9.50E+01 | 18% |
|  |  | *Penicillium* sp. | 2.94E+02 | 54% | 2.88E+02 | 32% | 3.62E+02 | 63% | 4.60E+01 | 9% |
|  |  | *Rhizopus* sp. | 1.23E+02 | 23% | 8.80E+01 | 10% | 1.20E+02 | 21% | 7.10E+01 | 14% |
|  | Manual | *A.* section *Nigri* | 1.60E+01 | 9% |  |  |  |  |  |  |
|  |  | *Cladosporium* sp. |  |  | 4.00E+00 | 10% |  |  | 4.00E+00 | 10% |
|  |  | *Mucor* sp. | 1.80E+01 | 10% |  |  | 4.00E+00 | 5% |  |  |
|  |  | *Penicillium* sp. | 1.33E+02 | 75% | 7.00E+00 | 18% | 1.10E+01 | 14% | 1.40E+01 | 34% |
|  |  | *Rhizomucor* sp. |  |  |  |  |  |  | 1.70E+01 | 41% |
|  |  | *Rhizopus* sp. | 1.00E+01 | 6% | 2.90E+01 | 73% | 6.20E+01 | 81% | 6.00E+00 | 15% |

Table S4 - Molecular detection of *Aspergillus* sections

| *Aspergillus* sections | Matrices | CFU.g^−1^/ CFU.m-^3^ (MEA/DG18) | C_t_ |
| --- | --- | --- | --- |
| *Aspergillus* section *Fumigati* | Settled dust | 0/0 | 27.65 |
|  |  | 0/0 | 26.47 |
|  |  | 0/0 | 29.19 |
|  |  | 0/0 | 33.41 |
|  | Filter | 0/0 | 35.75 |
| *Aspergillus* section *Circumdati* | Settled dust | 0/0 | 35.58 |
|  | Filter | 0/1.35 x 10^0^ | 35.88 |
|  | Filter | 0/0 | 13.37 |

C_t_ - Cycle threshold

Table S5 - Comparison of bacterial counts (TSA and VRBA), fungal counts (MEA and DG18) and azole resistance (SDA, ICZ, VCZ and PCZ) between seasons, in each sampling method. Mann-Whitney test results.

| Sample Method |  | Culture medium | Season | n | Ranks | | Test Statistics | |
| --- | --- | --- | --- | --- | --- | --- | --- | --- |
|  |  |  |  |  | Mean Rank | Sum of Ranks | Mann-Whitney U | p |
| Filter | Bacteria | TSA | Summer | 21 | 34.24 | 719.00 | 184.000 | 0.006* |
|  |  |  | Autumn | 32 | 22.25 | 712.00 |  |  |
|  |  |  | Total | 53 |  |  |  |  |
|  |  | VRBA | Summer | 21 | 35.88 | 753.50 | 149.500 | 0.000* |
|  |  |  | Autumn | 32 | 21.17 | 677.50 |  |  |
|  |  |  | Total | 53 |  |  |  |  |
|  | Fungi | MEA | Summer | 21 | 18.10 | 380.00 | 149.000 | 0.001* |
|  |  |  | Autumn | 32 | 32.84 | 1051.00 |  |  |
|  |  |  | Total | 53 |  |  |  |  |
|  |  | DG18 | Summer | 21 | 26.29 | 552.00 | 321.000 | 0.785 |
|  |  |  | Autumn | 32 | 27.47 | 879.00 |  |  |
|  |  |  | Total | 53 |  |  |  |  |
|  | Azole resistance | SDA | Summer | 21 | 28.48 | 598.00 | 305.000 | 0.573 |
|  |  |  | Autumn | 32 | 26.03 | 833.00 |  |  |
|  |  |  | Total | 53 |  |  |  |  |
|  |  | ICZ | Summer | 21 | 34.05 | 715.00 | 188.000 | 0.007* |
|  |  |  | Autumn | 32 | 22.38 | 716.00 |  |  |
|  |  |  | Total | 53 |  |  |  |  |
|  |  | VCZ | Summer | 21 | 35.05 | 736.00 | 167.000 | 0.002* |
|  |  |  | Autumn | 32 | 21.72 | 695.00 |  |  |
|  |  |  | Total | 53 |  |  |  |  |
|  |  | PCZ | Summer | 21 | 34.62 | 727.00 | 176.000 | 0.002* |
|  |  |  | Autumn | 32 | 22.00 | 704.00 |  |  |
|  |  |  | Total | 53 |  |  |  |  |
| Settled dust | Bacteria | TSA | Summer | 11 | 23.05 | 253.50 | 10.500 | 0.000* |
|  |  |  | Autumn | 18 | 10.08 | 181.50 |  |  |
|  |  |  | Total | 29 |  |  |  |  |
|  |  | VRBA | Summer | 11 | 23.05 | 253.50 | 10.500 | 0.000* |
|  |  |  | Autumn | 18 | 10.08 | 181.50 |  |  |
|  |  |  | Total | 29 |  |  |  |  |
|  | Fungi | MEA | Summer | 11 | 12.82 | 141.00 | 75.000 | 0.275 |
|  |  |  | Autumn | 18 | 16.33 | 294.00 |  |  |
|  |  |  | Total | 29 |  |  |  |  |
|  |  | DG18 | Summer | 11 | 18.18 | 200.00 | 64.000 | 0.114 |
|  |  |  | Autumn | 18 | 13.06 | 235.00 |  |  |
|  |  |  | Total | 29 |  |  |  |  |
|  | Azole resistance | SDA | Summer | 11 | 17.82 | 196.00 | 68.000 | 0.157 |
|  |  |  | Autumn | 18 | 13.28 | 239.00 |  |  |
|  |  |  | Total | 29 |  |  |  |  |
|  |  | ICZ | Summer | 11 | 22.82 | 251.00 | 13.000 | 0.000* |
|  |  |  | Autumn | 18 | 10.22 | 184.00 |  |  |
|  |  |  | Total | 29 |  |  |  |  |
|  |  | VCZ | Summer | 11 | 22.00 | 242.00 | 22.000 | 0.001* |
|  |  |  | Autumn | 18 | 10.72 | 193.00 |  |  |
|  |  |  | Total | 29 |  |  |  |  |
|  |  | PCZ | Summer | 11 | 19.68 | 216.50 | 47.500 | 0.017* |
|  |  |  | Autumn | 18 | 12.14 | 218.50 |  |  |
|  |  |  | Total | 29 |  |  |  |  |
| Swabs | Bacteria | TSA | Summer | 21 | 24.81 | 521.00 | 67.000 | 0.001* |
|  |  |  | Autumn | 17 | 12.94 | 220.00 |  |  |
|  |  |  | Total | 38 |  |  |  |  |
|  |  | VRBA | Summer | 21 | 21.19 | 445.00 | 143.000 | 0.144 |
|  |  |  | Autumn | 17 | 17.41 | 296.00 |  |  |
|  |  |  | Total | 38 |  |  |  |  |
|  | Fungi | MEA | Summer | 21 | 20.98 | 440.50 | 147.500 | 0.291 |
|  |  |  | Autumn | 17 | 17.68 | 300.50 |  |  |
|  |  |  | Total | 38 |  |  |  |  |
|  |  | DG18 | Summer | 21 | 20.83 | 437.50 | 150.500 | 0.247 |
|  |  |  | Autumn | 17 | 17.85 | 303.50 |  |  |
|  |  |  | Total | 38 |  |  |  |  |
| *Statistically significant differences | | | | | | | | |

Table S6 - Comparison of bacterial and fungal counts and resistance to azoles between industries, in summer (Mann-Whitney test results)

| Sample method |  | Culture Media | Industry | N | Ranks | | Test statistics | |
| --- | --- | --- | --- | --- | --- | --- | --- | --- |
|  |  |  |  |  | Mean Rank | Sum of Ranks | Mann-Whitney U | p |
| Filter | Bacteria | TSA | A | 16 | 8.88 | 142.00 | 6.000 | 0.005* |
|  |  |  | B | 5 | 17.80 | 89.00 |  |  |
|  |  |  | Total | 21 |  |  |  |  |
|  |  | VRBA | A | 16 | 9.44 | 151.00 | 15.000 | 0.035* |
|  |  |  | B | 5 | 16.00 | 80.00 |  |  |
|  |  |  | Total | 21 |  |  |  |  |
|  | Fungi | MEA | A | 16 | 11.69 | 187.00 | 29.000 | 0.364 |
|  |  |  | B | 5 | 8.80 | 44.00 |  |  |
|  |  |  | Total | 21 |  |  |  |  |
|  |  | DG18 | A | 16 | 11.69 | 187.00 | 29.000 | 0.364 |
|  |  |  | B | 5 | 8.80 | 44.00 |  |  |
|  |  |  | Total | 21 |  |  |  |  |
|  | Azole resistance | SDA | A | 16 | 11.88 | 190.00 | 26.000 | 0.248 |
|  |  |  | B | 5 | 8.20 | 41.00 |  |  |
|  |  |  | Total | 21 |  |  |  |  |
|  |  | ICZ | A | 16 | 12.66 | 202.50 | 13.500 | 0.029* |
|  |  |  | B | 5 | 5.70 | 28.50 |  |  |
|  |  |  | Total | 21 |  |  |  |  |
|  |  | VCZ | A | 16 | 12.38 | 198.00 | 18.000 | 0.069 |
|  |  |  | B | 5 | 6.60 | 33.00 |  |  |
|  |  |  | Total | 21 |  |  |  |  |
|  |  | PCZ | A | 16 | 10.56 | 169.00 | 33.000 | 0.562 |
|  |  |  | B | 5 | 12.40 | 62.00 |  |  |
|  |  |  | Total | 21 |  |  |  |  |
| Settled dust | Bacteria | TSA | A | 8 | 5.44 | 43.50 | 7.500 | 0.236 |
|  |  |  | B | 3 | 7.50 | 22.50 |  |  |
|  |  |  | Total | 11 |  |  |  |  |
|  |  | VRBA | A | 8 | 5.44 | 43.50 | 7.500 | 0.236 |
|  |  |  | B | 3 | 7.50 | 22.50 |  |  |
|  |  |  | Total | 11 |  |  |  |  |
|  | Fungi | MEA | A | 8 | 5.31 | 42.50 | 6.500 | 0.245 |
|  |  |  | B | 3 | 7.83 | 23.50 |  |  |
|  |  |  | Total | 11 |  |  |  |  |
|  |  | DG18 | A | 8 | 4.94 | 39.50 | 3.500 | 0.080 |
|  |  |  | B | 3 | 8.83 | 26.50 |  |  |
|  |  |  | Total | 11 |  |  |  |  |
|  | Azole resistance | SDA | A | 8 | 5.44 | 43.50 | 7.500 | 0.241 |
|  |  |  | B | 3 | 7.50 | 22.50 |  |  |
|  |  |  | Total | 11 |  |  |  |  |
|  |  | ICZ | A | 8 | 4.88 | 39.00 | 3.000 | 0.063 |
|  |  |  | B | 3 | 9.00 | 27.00 |  |  |
|  |  |  | Total | 11 |  |  |  |  |
|  |  | VCZ | A | 8 | 5.63 | 45.00 | 9.000 | 0.533 |
|  |  |  | B | 3 | 7.00 | 21.00 |  |  |
|  |  |  | Total | 11 |  |  |  |  |
|  |  | PCZ | A | 8 | 4.94 | 39.50 | 3.500 | 0.058 |
|  |  |  | B | 3 | 8.83 | 26.50 |  |  |
|  |  |  | Total | 11 |  |  |  |  |
| Swabs | Bacteria | TSA | A | 11 | 12.32 | 135.50 | 40.500 | 0.294 |
|  |  |  | B | 10 | 9.55 | 95.50 |  |  |
|  |  |  | Total | 21 |  |  |  |  |
|  |  | VRBA | A | 11 | 10.05 | 110.50 | 44.500 | 0.354 |
|  |  |  | B | 10 | 12.05 | 120.50 |  |  |
|  |  |  | Total | 21 |  |  |  |  |
|  | Fungi | MEA | A | 11 | 9.05 | 99.50 | 33.500 | 0.099 |
|  |  |  | B | 10 | 13.15 | 131.50 |  |  |
|  |  |  | Total | 21 |  |  |  |  |
|  |  | DG18 | A | 11 | 11.00 | 121.00 | 55.000 | 1.000 |
|  |  |  | B | 10 | 11.00 | 110.00 |  |  |
|  |  |  | Total | 21 |  |  |  |  |
| *Statistically significant differences | | | | | | | | |

Table S7 - Comparison of bacterial and fungal counts and resistance to azoles between industries, in autumn (Kruskal-Wallis and Mann-Whitney tests results)

| Sample method | Media | | Industry | n | Ranks | Test statistics | | |
| --- | --- | --- | --- | --- | --- | --- | --- | --- |
|  |  |  |  |  | Mean Rank | Kruskal-Wallis H or Mann-Whitney U | df | p |
| Filters | Bacteria | TSA | A | 9 | 10.83 | 12.799 | 5 | 0.025* |
|  |  |  | B | 3 | 27.00 |  |  |  |
|  |  |  | C | 5 | 21.50 |  |  |  |
|  |  |  | D | 6 | 10.83 |  |  |  |
|  |  |  | E | 3 | 15.00 |  |  |  |
|  |  |  | F | 6 | 22.00 |  |  |  |
|  |  |  | Total | 32 |  |  |  |  |
|  |  | VRBA | A | 9 | 16.22 | 18.444 | 5 | 0.002* |
|  |  |  | B | 3 | 13.00 |  |  |  |
|  |  |  | C | 5 | 13.00 |  |  |  |
|  |  |  | D | 6 | 26.83 |  |  |  |
|  |  |  | E | 3 | 13.00 |  |  |  |
|  |  |  | F | 6 | 13.00 |  |  |  |
|  |  |  | Total | 32 |  |  |  |  |
|  | Fungi | MEA | A | 9 | 9.78 | 19.562 | 5 | 0.002* |
|  |  |  | B | 3 | 22.00 |  |  |  |
|  |  |  | C | 5 | 27.00 |  |  |  |
|  |  |  | D | 6 | 9.00 |  |  |  |
|  |  |  | E | 3 | 27.00 |  |  |  |
|  |  |  | F | 6 | 17.33 |  |  |  |
|  |  |  | Total | 32 |  |  |  |  |
|  |  | DG18 | A | 9 | 14.78 | 8.111 | 5 | 0.150 |
|  |  |  | B | 3 | 8.33 |  |  |  |
|  |  |  | C | 5 | 17.00 |  |  |  |
|  |  |  | D | 6 | 14.33 |  |  |  |
|  |  |  | E | 3 | 28.33 |  |  |  |
|  |  |  | F | 6 | 19.00 |  |  |  |
|  |  |  | Total | 32 |  |  |  |  |
|  | Azole resistance | SDA | A | 9 | 9.22 | 20.344 | 5 | 0.001* |
|  |  |  | B | 3 | 18.00 |  |  |  |
|  |  |  | C | 5 | 27.00 |  |  |  |
|  |  |  | D | 6 | 9.83 |  |  |  |
|  |  |  | E | 3 | 29.00 |  |  |  |
|  |  |  | F | 6 | 18.33 |  |  |  |
|  |  |  | Total | 32 |  |  |  |  |
|  |  | ICCZ | A | 9 | 13.89 | 9.723 | 5 | 0.083 |
|  |  |  | B | 3 | 22.33 |  |  |  |
|  |  |  | C | 5 | 12.20 |  |  |  |
|  |  |  | D | 6 | 24.50 |  |  |  |
|  |  |  | E | 3 | 20.00 |  |  |  |
|  |  |  | F | 6 | 11.33 |  |  |  |
|  |  |  | Total | 32 |  |  |  |  |
|  |  | VCZ | A | 9 | 13.39 | 19.257 | 5 | 0.002* |
|  |  |  | B | 3 | 24.00 |  |  |  |
|  |  |  | C | 5 | 12.90 |  |  |  |
|  |  |  | D | 6 | 28.67 |  |  |  |
|  |  |  | E | 3 | 17.00 |  |  |  |
|  |  |  | F | 6 | 8.00 |  |  |  |
|  |  |  | Total | 32 |  |  |  |  |
|  |  | PCZ | A | 9 | 15.61 | 24.395 | 5 | 0.000* |
|  |  |  | B | 3 | 12.50 |  |  |  |
|  |  |  | C | 5 | 12.50 |  |  |  |
|  |  |  | D | 6 | 29.17 |  |  |  |
|  |  |  | E | 3 | 12.50 |  |  |  |
|  |  |  | F | 6 | 12.50 |  |  |  |
|  |  |  | Total | 32 |  |  |  |  |
| Settled dust | Bacteria | TSA | A | 4 | 15.00 | 11.043 | 3 | 0.011* |
|  |  |  | B | 4 | 6.50 |  |  |  |
|  |  |  | C | 4 | 5.13 |  |  |  |
|  |  |  | D | 6 | 10.75 |  |  |  |
|  |  |  | Total | 18 |  |  |  |  |
|  |  | VRBA | A | 4 | 15.00 | 9.438 | 3 | 0.024* |
|  |  |  | B | 4 | 5.75 |  |  |  |
|  |  |  | C | 4 | 6.13 |  |  |  |
|  |  |  | D | 6 | 10.58 |  |  |  |
|  |  |  | Total | 18 |  |  |  |  |
|  | Fungi | MEA | A | 4 | 3.50 | 7.400 | 3 | 0.060 |
|  |  |  | B | 4 | 12.25 |  |  |  |
|  |  |  | C | 4 | 12.13 |  |  |  |
|  |  |  | D | 6 | 9.92 |  |  |  |
|  |  |  | Total | 18 |  |  |  |  |
|  |  | DG18 | A | 4 | 8.25 | 0.729 | 3 | 0.866 |
|  |  |  | B | 4 | 10.25 |  |  |  |
|  |  |  | C | 4 | 8.38 |  |  |  |
|  |  |  | D | 6 | 10.58 |  |  |  |
|  |  |  | Total | 18 |  |  |  |  |
|  | Azole resistance | SDA | A | 4 | 8.63 | 11.753 | 3 | 0.008* |
|  |  |  | B | 4 | 2.50 |  |  |  |
|  |  |  | C | 4 | 14.75 |  |  |  |
|  |  |  | D | 6 | 11.25 |  |  |  |
|  |  |  | Total | 18 |  |  |  |  |
|  |  | ICZ | A | 4 | 12.50 | 4.214 | 3 | 0.239 |
|  |  |  | B | 4 | 8.50 |  |  |  |
|  |  |  | C | 4 | 5.63 |  |  |  |
|  |  |  | D | 6 | 10.75 |  |  |  |
|  |  |  | Total | 18 |  |  |  |  |
|  |  | VCZ | A | 4 | 13.00 | 4.867 | 3 | 0.182 |
|  |  |  | B | 4 | 8.50 |  |  |  |
|  |  |  | C | 4 | 5.25 |  |  |  |
|  |  |  | D | 6 | 10.67 |  |  |  |
|  |  |  | Total | 18 |  |  |  |  |
|  |  | PCZ | A | 4 | 14.88 | 10.027 | 3 | 0.018* |
|  |  |  | B | 4 | 6.00 |  |  |  |
|  |  |  | C | 4 | 6.00 |  |  |  |
|  |  |  | D | 6 | 10.58 |  |  |  |
|  |  |  | Total | 18 |  |  |  |  |
| Swabs | Bacteria | TSA | A | 8 | 9.94 | 12.500 |  | 0.065 |
|  |  |  | B | 7 | 5.79 |  |  |  |
|  |  |  | Total | 15 |  |  |  |  |
|  |  | VRBA | A | 8 | 8.88 | 21.000 |  | 0.171 |
|  |  |  | B | 7 | 7.00 |  |  |  |
|  |  |  | Total | 15 |  |  |  |  |
|  | Fungi | MEA | A | 8 | 7.13 | 21.000 |  | 0.298 |
|  |  |  | B | 7 | 9.00 |  |  |  |
|  |  |  | Total | 15 |  |  |  |  |
|  |  | DG18 | A | 8 | 7.00 | 20.000 |  | 0.118 |
|  |  |  | B | 7 | 9.14 |  |  |  |
|  |  |  | Total | 15 |  |  |  |  |
| *Statistically significant differences | | | | | | | | |

Table S8 - Comparison of bacterial, fungal and azole resistance counts between the automatic and manual industries assessed in the autumn. Results of Mann-Whitney test

| Sample method |  | Culture Media | Type of Workplaces assessed | N | Ranks | | Test statistics | |
| --- | --- | --- | --- | --- | --- | --- | --- | --- |
|  |  |  |  |  | Mean Rank | Sum of Ranks | Mann-Whitney U | p |
| Filters | Bacteria | TSA | Automated | 17 | 16.82 | 286.00 | 122.000 | 0.835 |
|  |  |  | Manual | 15 | 16.13 | 242.00 |  |  |
|  |  |  | Total | 32 |  |  |  |  |
|  |  | VRBA | Automated | 17 | 14.71 | 250.00 | 97.000 | 0.111 |
|  |  |  | Manual | 15 | 18.53 | 278.00 |  |  |
|  |  |  | Total | 32 |  |  |  |  |
|  | Fungi | MEA | Automated | 17 | 17.00 | 289.00 | 119.000 | 0.748 |
|  |  |  | Manual | 15 | 15.93 | 239.00 |  |  |
|  |  |  | Total | 32 |  |  |  |  |
|  |  | DG18 | Automated | 17 | 14.29 | 243.00 | 90.000 | 0.157 |
|  |  |  | Manual | 15 | 19.00 | 285.00 |  |  |
|  |  |  | Total | 32 |  |  |  |  |
|  | Azole resistance | SDA | Automated | 17 | 16.00 | 272.00 | 119.000 | 0.748 |
|  |  |  | Manual | 15 | 17.07 | 256.00 |  |  |
|  |  |  | Total | 32 |  |  |  |  |
|  |  | ICZ | Automated | 17 | 14.88 | 253.00 | 100.000 | 0.294 |
|  |  |  | Manual | 15 | 18.33 | 275.00 |  |  |
|  |  |  | Total | 32 |  |  |  |  |
|  |  | VCZ | Automated | 17 | 15.12 | 257.00 | 104.000 | 0.367 |
|  |  |  | Manual | 15 | 18.07 | 271.00 |  |  |
|  |  |  | Total | 32 |  |  |  |  |
|  |  | PCZ | Automated | 17 | 14.15 | 240.50 | 87.500 | 0.047* |
|  |  |  | Manual | 15 | 19.17 | 287.50 |  |  |
|  |  |  | Total | 32 |  |  |  |  |
| Settled dust | Bacteria | TSA | Automated | 12 | 8.88 | 106.50 | 28.500 | 0.424 |
|  |  |  | Manual | 6 | 10.75 | 64.50 |  |  |
|  |  |  | Total | 18 |  |  |  |  |
|  |  | VRBA | Automated | 12 | 8.96 | 107.50 | 29.500 | 0.510 |
|  |  |  | Manual | 6 | 10.58 | 63.50 |  |  |
|  |  |  | Total | 18 |  |  |  |  |
|  | Fungi | MEA | Automated | 12 | 9.29 | 111.50 | 33.500 | 0.811 |
|  |  |  | Manual | 6 | 9.92 | 59.50 |  |  |
|  |  |  | Total | 18 |  |  |  |  |
|  |  | DG18 | Automated | 12 | 8.96 | 107.50 | 29.500 | 0.541 |
|  |  |  | Manual | 6 | 10.58 | 63.50 |  |  |
|  |  |  | Total | 18 |  |  |  |  |
|  | Azole resistance | SDA | Automated | 12 | 8.63 | 103.50 | 25.500 | 0.320 |
|  |  |  | Manual | 6 | 11.25 | 67.50 |  |  |
|  |  |  | Total | 18 |  |  |  |  |
|  |  | ICZ | Automated | 12 | 8.88 | 106.50 | 28.500 | 0.462 |
|  |  |  | Manual | 6 | 10.75 | 64.50 |  |  |
|  |  |  | Total | 18 |  |  |  |  |
|  |  | VCZ | Automated | 12 | 8.92 | 107.00 | 29.000 | 0.504 |
|  |  |  | Manual | 6 | 10.67 | 64.00 |  |  |
|  |  |  | Total | 18 |  |  |  |  |
|  |  | PCZ | Automated | 12 | 8.96 | 107.50 | 29.500 | 0.488 |
|  |  |  | Manual | 6 | 10.58 | 63.50 |  |  |
|  |  |  | Total | 18 |  |  |  |  |
| *Statistically significant differences | | | | | | | | |

Table S9 – Comparison of culture media (results from Wilcoxon and Friedman tests)

| Sample method |  | Culture media |  | n | Ranks | | Test statistics | | |
| --- | --- | --- | --- | --- | --- | --- | --- | --- | --- |
|  |  |  |  |  | Mean Rank | Sum of Ranks | z | df | p |
| Filter | Bacteria | VRBA - TSA | Negative Ranks | 33^a^ | 23.73 | 783.00 | -2.318^g^ |  | 0.020* |
|  |  |  | Positive Ranks | 14^b^ | 24.64 | 345.00 |  |  |  |
|  |  |  | Ties | 6^c^ |  |  |  |  |  |
|  |  |  | Total | 53 |  |  |  |  |  |
|  | Fungi | DG18 - MEA | Negative Ranks | 24^d^ | 24.21 | 581.00 | -0.072^h^ |  | 0.943 |
|  |  |  | Positive Ranks | 24^e^ | 24.79 | 595.00 |  |  |  |
|  |  |  | Ties | 5^f^ |  |  |  |  |  |
|  |  |  | Total | 53 |  |  |  |  |  |
|  | Azole resistance | SDA  ICZ  VCZ  PCZ | | 53 | 2.98 |  | 36.661^i^ | 3 | 0.000* |
|  |  |  |  |  | 2.53 |  |  |  |  |
|  |  |  |  |  | 2.83 |  |  |  |  |
|  |  |  |  |  | 1.66 |  |  |  |  |
| Settled dust | Bacteria | VRBA - TSA | Negative Ranks | 15^a^ | 9.00 | 135.00 | -3.549^g^ |  | 0.000* |
|  |  |  | Positive Ranks | 1^b^ | 1.00 | 1.00 |  |  |  |
|  |  |  | Ties | 13^c^ |  |  |  |  |  |
|  |  |  | Total | 29 |  |  |  |  |  |
|  | Fungi | DG18 - MEA | Negative Ranks | 10^d^ | 19.60 | 196.00 | -0.160^h^ |  | 0.873 |
|  |  |  | Positive Ranks | 18^e^ | 11.67 | 210.00 |  |  |  |
|  |  |  | Ties | 1^f^ |  |  |  |  |  |
|  |  |  | Total | 29 |  |  |  |  |  |
|  | Azole resistance | SDA | | 29 | 2.66 |  | 3.163^i^ |  | 0.367 |
|  |  | ICZ | |  | 2.48 |  |  |  |  |
|  |  | VCZ | |  | 2.67 |  |  |  |  |
|  |  | PCZ | |  | 2.19 |  |  |  |  |
| Swabs | Bacteria | VRBA - TSA | Negative Ranks | 33^a^ | 18.55 | 612.00 | -3.933^g^ |  | 0.000* |
|  |  |  | Positive Ranks | 4^b^ | 22.75 | 91.00 |  |  |  |
|  |  |  | Ties | 1^c^ |  |  |  |  |  |
|  |  |  | Total | 38 |  |  |  |  |  |
|  | Fungi | DG18 - MEA | Negative Ranks | 13^d^ | 9.85 | 128.00 | -2.480^g^ |  | 0.013* |
|  |  |  | Positive Ranks | 4^e^ | 6.25 | 25.00 |  |  |  |
|  |  |  | Ties | 21^f^ |  |  |  |  |  |
|  |  |  | Total | 38 |  |  |  |  |  |
| a. VRBA < TSA. b. VRBA > TSA. c. VRBA = TSA. d. DG18 < MEA. e. DG18 > MEA. f. DG18 = MEA. g. Wilcoxon Signed Ranks Test, based on positive ranks. h. Wilcoxon Signed Ranks Test, based on negative ranks. i. Friedman test. *Statistically significant differences | | | | | | | | | |

Table S10 - Shannon and Simpson indexes to assess species diversity.

| Type of Workplaces assessed | Sample method | Industry | Culture media | Species | Culture media (CFU/m-2) | Shannon index (H) | Simpson index (D) |
| --- | --- | --- | --- | --- | --- | --- | --- |
| Automated | Filters | A | MEA | *Aspergillus* section *Nigri* | 21.54 | 0.27 | 1.16 |
|  |  |  |  | *Penicillium* sp. | 263.52 |  |  |
|  |  |  | DG18 | *Penicillium* sp. | 1364.24 | 0.09 | 1.03 |
|  |  |  |  | *Aspergillus* section *Circumdati* | 15.96 |  |  |
|  |  |  |  | *Aspergillus* section *Aspergilli* | 1.19 |  |  |
|  |  |  |  | *Aspergillus* section *Fumigati* | 4.52 |  |  |
|  |  | B | MEA | *Cladosporium sp.* | 1.37 | 1.01 | 2.48 |
|  |  |  |  | *Aspergillus* section *Nigri* | 40.04 |  |  |
|  |  |  |  | *Penicillium* sp. | 136.02 |  |  |
|  |  |  |  | *Rhizopus* sp. | 75.95 |  |  |
|  |  |  | DG18 | *Aspergillus* section *Circumdati* | 2.54 | 0.13 | 1.06 |
|  |  |  |  | *Penicillium* sp. | 87.02 |  |  |
|  |  | C | MEA | *Aspergillus* section *Flavi* | 2.27 | 0.82 | 1.90 |
|  |  |  |  | *Aspergillus* section *Nigri* | 66.87 |  |  |
|  |  |  |  | *Penicillium* sp. | 546.67 |  |  |
|  |  |  |  | *Rhizopus* sp. | 184.39 |  |  |
|  |  |  | DG18 | *Aspergillus* section *Aspergilli* | 2.27 | 0.95 | 1.83 |
|  |  |  |  | *Aspergillus* section *Circumdati* | 13.85 |  |  |
|  |  |  |  | *Cladosporium* sp. | 1.14 |  |  |
|  |  |  |  | *Aspergillus* section *Flavi* | 15.59 |  |  |
|  |  |  |  | *Aspergillus* section *Nigri* | 6.79 |  |  |
|  |  |  |  | *Penicillium* sp. | 103.57 |  |  |
|  | Settled dust | A | MEA | *Mucro* sp*.* | 13.00 | 0.97 | 2.11 |
|  |  |  |  | *Aspergillus* section *Nigri* | 43.00 |  |  |
|  |  |  |  | *Penicillium* sp. | 118.00 |  |  |
|  |  |  |  | *Rhizopus* sp. | 10.00 |  |  |
|  |  |  | DG18 | *Aspergillus* section *Aspergilli* | 26.00 | 0.84 | 1.62 |
|  |  |  |  | *Aspergillus* section *Circumdati* | 14.00 |  |  |
|  |  |  |  | *Aspergillus* section *Flavi* | 1.00 |  |  |
|  |  |  |  | *Aspergillus* section *Fumigati* | 26.00 |  |  |
|  |  |  |  | *Aspergillus* section *Nigri* | 13.00 |  |  |
|  |  |  |  | *Penicillium* sp. | 279.00 |  |  |
|  |  | B | MEA | *Aspergillus* section *Nidulantes* | 9.00 | 0.96 | 1.87 |
|  |  |  |  | *Aspergillus* section *Nigri* | 27.00 |  |  |
|  |  |  |  | *Penicillium* sp. | 134.00 |  |  |
|  |  |  |  | *Rhizopus* sp. | 9.00 |  |  |
|  |  |  |  | *S. racemosum* | 9.00 |  |  |
|  |  |  | DG18 | *Aspergillus* section *Nigri* | 7.00 | 0.50 | 1.10 |
|  |  |  |  | *Penicillium* sp. | 132.00 |  |  |
|  |  |  |  | *Cladosporium* sp. | 1.00 |  |  |
|  |  |  |  | *Aspergillus* section *Flavi* | 18.00 |  |  |
|  |  | C | MEA | *Aspergillus* section *Nidulantes* | 1.00 | 0.56 | 1.41 |
|  |  |  |  | *Aspergillus* section *Nigri* | 29.00 |  |  |
|  |  |  |  | *Penicillium* sp. | 192.00 |  |  |
|  |  |  |  | *Rhizopus* sp. | 9.00 |  |  |
|  |  |  | DG18 | *Aspergillus* section *Aspergilli* | 1.00 | 0.96 | 2.05 |
|  |  |  |  | *Aspergillus* section *Circumdati* | 16.00 |  |  |
|  |  |  |  | *Aspergillus* section *Flavi* | 9.00 |  |  |
|  |  |  |  | *Aspergillus* section *Nigri* | 1.00 |  |  |
|  |  |  |  | *Penicillium* sp. | 52.00 |  |  |
|  | EDC | A | DG18 | *Penicillium* sp. | 7.58 |  |  |
|  | eSwabs Exposed | A | MEA | *Penicillium* sp. | 10000.00 |  |  |
|  |  |  | DG18 | *Cladosporium* sp. | 2000.00 | 0.56 | 1.60 |
|  |  |  |  | *Penicillium* sp. | 6000.00 |  |  |
|  | eSwabs Control | A | MEA | *Cladosporium* sp. | 4000.00 | 0.64 | 1.80 |
|  |  |  |  | *Penicillium* sp. | 2000.00 |  |  |
|  | eSwabs Exposed | B | MEA | *Chrysosporium* sp. | 2000.00 | 0.87 | 2.00 |
|  |  |  |  | *Aspergillus* section Fumigati | 2000.00 |  |  |
|  |  |  |  | *Penicillium* sp. | 8000.00 |  |  |
|  |  |  | DG18 | *Alternaria* sp. | 2000.00 | 1.34 | 2.95 |
|  |  |  |  | *C. sitophila* | 2000.00 |  |  |
|  |  |  |  | *Cladosporium* sp. | 12000.00 |  |  |
|  |  |  |  | *Aspergillus* section *Nigri* | 4000.00 |  |  |
|  |  |  |  | *Penicillium* sp. | 22000.00 |  |  |
|  |  |  |  | *Phoma* sp. | 2000.00 |  |  |
|  | eSwabs Control | C | DG18 | *Penicillium* sp. | 6000.00 |  |  |
| Manual | Filters | D | MEA | *Cladosporium* sp. | 2.11 | 0.46 | 1.25 |
|  |  |  |  | *Aspergillus* section *Nigri* | 3.17 |  |  |
|  |  |  |  | *Penicillium* sp. | 69.71 |  |  |
|  |  |  |  | *Rhizopus* sp. | 3.16 |  |  |
|  |  |  | DG18 | *Mucro* sp. | 5.29 | 0.16 | 1.07 |
|  |  |  |  | *Aspergillus* section *Nidulantes* | 1.08 |  |  |
|  |  |  |  | *Penicillium* sp. | 194.38 |  |  |
|  |  | E | MEA | *Aspergillus* section *Nigri* | 4.44 | 0.21 | 1.10 |
|  |  |  |  | *Penicillium* sp. | 447.95 |  |  |
|  |  |  |  | *Rhizopus* sp. | 17.78 |  |  |
|  |  |  | DG18 | *Aspergillus* section *Aspergilli* | 5.56 | 0.14 | 1.05 |
|  |  |  |  | *Aspergillus* section *Circumdati* | 1.11 |  |  |
|  |  |  |  | *Penicillium* sp. | 330.68 |  |  |
|  |  |  |  | *S. racemosum* | 2.22 |  |  |
|  |  | F | MEA | *Aspergillus* section *Fumigati* | 18.91 | 0.31 | 1.15 |
|  |  |  |  | *Aspergillus* section *Nigri* | 8.97 |  |  |
|  |  |  |  | *Paecilomyces* sp. | 1.13 |  |  |
|  |  |  |  | *Penicillium* sp. | 387.63 |  |  |
|  |  |  | DG18 | *Aspergillus* section *Circumdati* | 8.24 | 0.17 | 1.07 |
|  |  |  |  | *Aspergillus* section *Flavi* | 2.11 |  |  |
|  |  |  |  | *Aspergillus* section *Nidulantes* | 1.96 |  |  |
|  |  |  |  | *Penicillium* sp. | 357.52 |  |  |
|  | Settled dust | D | MEA | *Aspergillus* section *Nigri* | 27.00 | 0.64 | 1.44 |
|  |  |  |  | *Paecilomyces* sp. | 11.00 |  |  |
|  |  |  |  | *Penicillium* sp. | 227.00 |  |  |
|  |  |  |  | *Rhizopus* sp. | 10.00 |  |  |
|  |  |  | DG18 | *Aspergillus* section *Circumdati* | 1.00 | 0.72 | 1.45 |
|  |  |  |  | *Cladosporium* sp. | 8.00 |  |  |
|  |  |  |  | *Aspergillus* section *Flavi* | 3.00 |  |  |
|  |  |  |  | *Aspergillus* section *Fumigati* | 4.00 |  |  |
|  |  |  |  | *Aspergillus* section *Nigri* | 5.00 |  |  |
|  |  |  |  | *Penicillium* sp. | 99.00 |  |  |
|  | EDC | D | MEA | *Aspergillus* section *Nidulantes* | 30.33 | 0.67 | 1.92 |
|  |  |  |  | *Penicillium* sp. | 45.50 |  |  |
|  |  |  | DG18 | *Penicillium* sp. | 121.32 |  |  |

# Supplementary text:

**Text S1: Full text of the interpretation of table 5 of the article**.

Concerning the sampling method, and in particular the filters in TSA, presented significant correlations: of weak intensity, with SDA counts (rS=0.374, p=0.006), revealing that higher bacterial counts found in filters in TSA is related to higher values in SDA. Bacterial counts in VRBA with fungal counts in MEA (rS=-0.563, p<0.0001), in VCZ (rS=0.448, p=0.001) and in PCZ (rS=0.528, p<0.0001), revealing that higher bacterial counts in VRBA is related to lower fungal counts in MEA and higher values in VCZ and PCZ. Fungal counts in MEA with fungal counts in DG18 (rS=0.373, p=0.006), counts in SDA (rS=0.582, p<0.0001), in VCZ (rS=-0.304, p=0.027) and in PCZ (rS=-0.483, p0.0001), indicating that a higher counts in MEA is related to higher counts in DG18 and SDA and lower values in VCZ and PCZ. Fungal counts in DG18 with SDA (rS=0.382, p=0.005), indicating that higher counts in DG18 is related to higher values in SDA. Concerning to azoles resistance, significant correlations were detected, of strong intensity, between ICZ and VCZ (rS=0.745, p<0.0001) and PCZ (rS=0.648, p<0.0001) and between VCZ and PCZ (rS=0.726, p<0.0001), showing that higher values in a given culture medium are related to higher values in another (Table 5).

In settled dust, considering the counts found in TSA, significant correlations were detected, with intensity ranging from weak to strong, with the VRBA counts (rS=0.979, p<0.0001), MEA counts (rS= - 0.470, p=0.010), ICZ counts (rS=0.918, p<0.0001), VCZ counts (rS=0.791, p<0.0001) and PCZ counts (rS=0.807, p<0.0001), revealing that higher bacterial counts in TSA is related with higher counts in VRBA, lower counts on MEA and higher counts in ICZ, VCZ and PCZ. Significant correlations between VRBA counts and fungal counts in MEA (rS= - 0.487, p=0.007), ICZ (rS=0.932, p<0.0001), VCZ (rS=0.764, p<0.0001) and PCZ (rS=0.799, p<0.0001), showing that higher bacterial counts in VRBA is related with lower fungal counts in MEA and higher values in ICZ, VCZ and PCZ. Significant correlations between fungal counts in MEA and in ICZ (rS= - 0.404, p=0.030), VCZ (rS= - 0.431, p=0.020) and PCZ (rS= - 0.625, p<0.0001), indicating that higher fungal counts in MEA is related with lower values counts in ICZ, VCZ and PCZ. Concerning counts in ICZ, significant correlations were detected with VCZ (rS=0.793, p<0.0001) and PCZ (rS=0.784, p<0.0001), revealing that higher values in ICZ are related with higher values in VCZ and PCZ. Azoles resistance in VCZ is correlated with PCZ (rS=0.658, p<0.0001), indicating that higher values in VCZ are related with higher values in PCZ (Table 5).

Considering the surfaces eSwabs sampling method, only a significant correlation, but with weak intensity, was detected between the bacterial counts in the TSA and in VRBA (rS=0.332, p=0.042), indicating that higher bacterial counts in TSA is related with higher counts in VRBA (Table 5).
